# Effects of the I559P gp41 Change on the Conformation and Function of the Human Immunodeficiency Virus (HIV-1) Membrane Envelope Glycoprotein Trimer

**DOI:** 10.1371/journal.pone.0122111

**Published:** 2015-04-07

**Authors:** Nirmin Alsahafi, Olfa Debbeche, Joseph Sodroski, Andrés Finzi

**Affiliations:** 1 Department of Microbiology and Immunology, McGill University, Montreal, H3A 2B4 Quebec, Canada; 2 Centre de Recherche du Centre Hospitalier de l'Université de Montréal, Montréal, H2X 0A9, Québec, Canada; 3 Department of Microbiology, Infectiology and Immunology, Université de Montréal, Montréal, H3C 3J7 Quebec, Canada; 4 Department of Cancer Immunology and AIDS, Dana-Farber Cancer Institute, Boston, Massachusetts, 02115, United States of America; 5 Department of Microbiology & Immunobiology, Harvard Medical School, Boston, Massachusetts, 02115, United States of America; 6 Department of Immunology and Infectious Diseases, Harvard School of Public Health, Boston, Massachusetts, 02115, United States of America; Lady Davis Institute for Medical Research, CANADA

## Abstract

The mature human immunodeficiency virus (HIV-1) envelope glycoprotein (Env) trimer is produced by proteolytic cleavage of a precursor and consists of three gp120 exterior and three gp41 transmembrane subunits. The metastable Env complex is induced to undergo conformational changes required for virus entry by the binding of gp120 to the receptors, CD4 and CCR5/CXCR4. An isoleucine-to-proline change (I559P) in the gp41 ectodomain has been used to stabilize soluble forms of HIV-1 Env trimers for structural characterization and for use as immunogens. In the native membrane-anchored HIV-1_BG505_ Env, the I559P change modestly decreased proteolytic maturation, increased the non-covalent association of gp120 with the Env trimer, and resulted in an Env conformation distinctly different from that of the wild-type HIV-1_BG505_ Env. Compared with the wild-type Env, the I559P Env was recognized inefficiently by polyclonal sera from HIV-1-infected individuals, by several gp41-directed antibodies, by some antibodies against the CD4-binding site of gp120, and by antibodies that preferentially recognize the CD4-bound Env. Some of the gp120-associated antigenic differences between the wild-type HIV-1_BG505_ Env and the I559P mutant were compensated by the SOS disulfide bond between gp120 and gp41, which has been used to stabilize cleaved soluble Env trimers. Nonetheless, regardless of the presence of the SOS changes, Envs with proline 559 were recognized less efficiently than Envs with isoleucine 559 by the VRC01 neutralizing antibody, which binds the CD4-binding site of gp120, and the PGT151 neutralizing antibody, which binds a hybrid gp120-gp41 epitope. The I559P change completely eliminated the ability of the HIV-1_BG505_ Env to mediate cell-cell fusion and virus entry, and abolished the capacity of the SOS Env to support virus infection in the presence of a reducing agent. These results suggest that differences exist between the quaternary structures of functional Env spikes and I559P Envs.

## Introduction

Human immunodeficiency virus (HIV-1) entry into the host cell is mediated by the viral envelope glycoproteins (Envs), which are derived by proteolytic cleavage of a trimeric gp160 Env precursor [1–3]. The resulting mature Env complex is composed of three gp120 exterior subunits and three gp41 transmembrane subunits. The metastable Env trimer undergoes conformational changes upon binding of gp120 to the receptors, CD4 and CCR5/CXCR4, that promote the fusion of the viral and target cell membranes by gp41 [1,4–17]. CD4 binding results in the pre-hairpin intermediate, in which the gp41 heptad repeat 1 (HR1) α-helical coiled coil is formed and exposed [18–22]. The binding of gp120 to either CCR5 or CXCR4 is thought to trigger the formation of a stable gp41 six-helix bundle required for fusion of the viral and target cell membranes [4–7,18–20,22–24].

Metastability and membrane localization are essential for HIV-1 Env function, but create difficulties for the preparation of stable, homogeneous Envs for structural analysis. Various approaches have been used to produce tractable soluble Env trimers lacking the transmembrane anchor and cytoplasmic tail [25–34]. Soluble gp140 Envs typically exhibit multiple oligomeric species in solution, indicating the weakness of the interprotomer contacts in the HIV-1 Env ectodomain and motivating efforts to improve their stability [25–34]. Proteolytically processed versions of soluble gp140 Env have been produced by the introduction of an artificial disulfide bond (SOS) linking the gp120 and gp41 subunits [31–34]. To attempt to destabilize the pre-hairpin intermediate and produce Envs in the mature, unliganded state, the helix-breaking isoleucine-to-proline change (I559P) has been introduced into the gp41 ectodomain [30]. The I559P change was found to stabilize soluble SOS gp140 trimers, and the I559P alteration has been combined with the SOS disulfide bond and deletion of the membrane-proximal gp41 region to produce soluble gp140 SOSIP.664 glycoproteins [34,35]. X-ray and cryoelectron microscopy (cryo-EM) structures of these soluble gp140 SOSIP glycoproteins bound to Fab fragments of neutralizing antibodies have been solved [36–39]. These structures provide information on the nature and arrangement of neutralizing antibody epitopes on soluble HIV-1 Env trimers. However, the soluble gp140 SOSIP.664 structures differ from cryo-EM maps of unliganded, proteolytically mature Env trimers derived from virions or uncleaved Env trimers from cell surfaces [40–43]. Moreover, the structure of the gp120 core (gp120 lacking the V1/V2 and V3 variable regions and N/C termini) in the soluble gp140 SOSIP.664 Env crystals unexpectedly resembles the CD4-bound conformation,[36–38,44]. These observations suggest possible differences in conformation between the soluble gp140 SOSIP.664 trimers and the unliganded functional Env spike on the surface of infected cells and virions. Here we investigate the impact of the I559P change on the conformation and function of the membrane-anchored Env from the primary HIV-1_BG505_ strain, from which the crystallized SOSIP.664 Env trimers [37,38] were derived. We also investigate the conformation of the gp120 core in the HIV-1_BG505_ Env by studying the phenotypes of a mutant, S375W, that predisposes gp120 to assume the CD4-bound state.

## Materials and Methods

### Cell lines

293T human embryonic kidney, Cf2Th canine thymocytes (American Type Culture Collection), HOS and TZM-bl cell lines (NIH AIDS Research and Reference Reagent Program) were grown at 37°C and 5% CO_2_ in Dulbecco’s modified Eagle’s medium (Invitrogen) containing 10% fetal bovine serum (Sigma) and 100 μg/ml of penicillin-streptomycin (Mediatech). Cf2Th cells stably expressing human CD4 and CCR5 [45] were grown in medium supplemented with 0.4 mg/ml of G418 (Invitrogen) and 0.15 mg/ml of hygromycin B (Roche Diagnostics). The TZM-bl cell line is a HeLa cell line stably expressing high levels of CD4 and CCR5 and possessing an integrated copy of the luciferase gene under control of the HIV-1 long terminal repeat [46].

### Site-directed mutagenesis

The sequence of full-length clade A HIV-1_BG505_ Env [47] was codon-optimized (GenScript) and cloned into the expression plasmid pcDNA3.1. Mutations were introduced individually or in combination into this plasmid, pcDNA3.1-BG505. Site-directed mutagenesis was performed using the QuikChange II XL site-directed mutagenesis protocol (Stratagene). For cell-based ELISA experiments, a stop codon was introduced to replace the codon for Gly 711, truncating the cytoplasmic tail (ΔCT) and enhancing cell-surface expression of selected HIV-1_BG505_ Env variants. The presence of the desired mutations was determined by automated DNA sequencing. The numbering of the HIV-1 Env amino acid residues is based on that of the prototypic HXBc2 strain of HIV-1, where position 1 is the initial methionine [48].

### Immunoprecipitation of envelope glycoproteins

For pulse-labeling experiments, 3X10^5^ 293T cells were cotransfected by the calcium phosphate method with the pcDNA3.1-BG505 vector expressing wild-type or mutant envelope glycoproteins. One day after transfection, cells were metabolically labeled for 5 h with 100 μCi/mL [^35^S]methionine-cysteine ([^35^S] Protein Labeling Mix; Perkin-Elmer) in Dulbecco’s modified Eagle’s medium lacking methionine and cysteine and supplemented with 5% dialyzed fetal bovine serum and then chased over-night with complete DMEM medium containing excess of methionine and cysteine. Cells were subsequently lysed in RIPA buffer (140 mM NaCl, 8 mM Na_2_HPO_4_, 2 mM NaH_2_PO_4_, 1% NP40, 0.05% sodium dodecyl sulfate (SDS)). Precipitation of radiolabeled HIV-1_BG505_ envelope glycoproteins from cell lysates or medium was performed with a mixture of sera from HIV-1-infected individuals for 1 hour at 4°C in the presence of 50 μl of 10% Protein A-Sepharose (American BioSciences). Written informed consent was obtained from all study participants, research adhered to the ethical guidelines of CRCHUM and was reviewed and approved by CRCHUM institutional review board (ethics committee). Sera was collected during Ficoll isolation of PBMCs and conserved at -80°C. Sera aliquots were heat-inactivated for 30 min at 56°C and stored at 4°C until ready to use in subsequent experiments.

Processing and association indices were determined by precipitation of radiolabeled cell lysates and supernatants with mixtures of sera from HIV-1-infected individuals. The association index is a measure of the ability of the mutant gp120 molecule to remain associated with the Env trimer complex on the expressing cell, relative to that of the wild-type Env trimers. The association index is calculated as follows: association index = ([mutant gp120]_cell_ × [wild-type gp120]_supernatant_)/ ([mutant gp120]_supernatant_ × [wild-type gp120]_cell_). The processing index is a measure of the conversion of the mutant gp160 Env precursor to mature gp120, relative to that of the wild-type Env trimers. The processing index was calculated by the formula: processing index = ([total gp120]_mutant_ × [gp160]_wild-type_)/ ([gp160]_mutant_ × [total gp120]_wild-type_).

### Cell-based ELISA

Detection of trimeric Env on the surface of HOS cells was performed by cell-based ELISA, as described [49,50]. Briefly, HOS cells were seeded in 96-well plates (2x10^4^ cells per well) and transfected the next day with 150 ng of ΔCT or 250 ng of full-length (gp160) Env-expressing plasmids using the standard polyethylenimine (PEI, Polyscience Inc, PA, USA) transfection method. Two days later, cells were washed twice with blocking buffer (10 mg/ml non-fat dry milk, 1.8 mM CaCl_2_, 1 mM MgCl_2_, 25 mM Tris,pH 7.5 and 140 mM NaCl) and then incubated for 1 h at room temperature with 20 nM CD4-Ig or anti-HIV-1 Env monoclonal antibodies recognizing CD4-induced gp120 epitopes (17b, A32), CD4-binding site (CD4BS) gp120 epitopes (VRC01, b12), gp120 V2 glycans (PG9), the gp120 V3 region (19b, GE2 JG8), gp120 V3 glycans (PGT121), gp120 outer domain glycans (2G12), the gp120-gp41 interface (PGT151, 35O22), and gp41 Cluster I (F240, 7B.2), Cluster II (2.2B) and MPER (4e10) epitopes. When indicated, cells were pre-incubated for 40 min at room temperature in absence or presence of 80 nM sCD4 before adding the first antibody. All ligands were diluted in blocking buffer. A horseradish peroxidase-conjugated antibody specific for the Fc region of human IgG (Pierce) was then incubated with the samples for 45 minutes at room temperature. For all conditions, cells were washed 5 times with blocking buffer and 5 times with washing buffer. HRP enzyme activity was determined after the addition of 30 μl per well of a 1:1 mix of Western Lightning oxidizing and luminol reagents (Perkin Elmer Life Sciences). Light emission was measured with an LB 941 TriStar luminometer (Berthold Technologies).

### Single-round luciferase-expressing viruses

Single-round viruses containing the firefly luciferase gene were produced by calcium phosphate transfection of 293T cells with the HIV-1 proviral vector pNL4.3Env^–^Luc and the pcDNA3.1 BG505 plasmid expressing the wild-type or mutant HIV-1_BG505_ envelope glycoproteins at a ratio of 2:1. Two days after transfection, the cell supernatants were harvested; the reverse transcriptase activities of all viruses were measured as described previously [51]. The virus-containing supernatants were stored in aliquots at -80°C.

### Infection by single-round luciferase-expressing viruses

Cf2Th-CD4/CCR5 target cells were seeded at a density of 5 X 10^3^ cells/well in 96-well luminometer-compatible tissue culture plates (Corning) 24 h before infection. Normalized amounts of viruses (as evaluated by reverse transcriptase activity of the viral stocks) were then added to the target cells followed by spin infection at 800 g for 1 h in 96-well plates at 25°C. When indicated, several concentrations (0.5, 1, 2.5 and 5 mM) of dithiothreitol (DTT) were added to the cells after the spin infection. In all cases, cells were incubated after the spin infection for 48 h at 37°C; the medium was then removed from each well, and the cells were lysed by the addition of 30 μl of passive lysis buffer (Promega) and three freeze-thaw cycles. An LB 941 TriStar luminometer (Berthold Technologies) was used to measure the luciferase activity of each well after the addition of 100 μl of luciferin buffer (15 mM MgSO_4_, 15 mM KPO_4_ [pH 7.8], 1 mM ATP, and 1 mM dithiothreitol) and 50 μl of 1 mM D-luciferin potassium salt (Prolume).

### Cell-cell fusion

To assess cell-to-cell fusion, 3 X 10^5^ 293T cells were cotransfected by the calcium phosphate method with an HIV-1 Tat-expressing plasmid, pLTR-Tat, and the pcDNA3.1 BG505 vector expressing the HIV-1_BG505_ envelope glycoprotein variants. Sixteen hours later, the cells were washed with fresh DMEM. Two days after transfection, the 293T cells were removed from the plate and counted. Then 3 X 10^4^ 293T cells were added to TZM-bl target cells that were seeded at a density of 2 X 10^4^ cells/well in 96-well luminometer-compatible tissue culture plates (Corning) 24 hours before the assay. When indicated, several concentrations (0.5, 1, 2.5 and 5 mM) of DTT were added to the cells after 30 min of co-incubation at room temperature and before incubation at 37°C. After six hours at 37°C, cells were lysed by the addition of 30 μl passive lysis buffer (Promega) and three freeze-thaw cycles. Luciferase activity in each well was measured as described above.

## Results

### Effect of the I559P and S375W changes on the function, processing and subunit association of the HIV-1_BG505_ Envs

In this study, the phenotypes of the I559P mutant were evaluated in the background of the wild-type (wt) HIV-1_BG505_ Env and in an Env with the SOS cysteine substitutions [52,53]. Because the structure of the gp120 core in the soluble gp140 SOSIP.664 Env crystals unexpectedly resembles the CD4-bound conformation [36–38,44], we also studied the S375W mutant, in which the aromatic ring of a tryptophan residue fills the Phe43 cavity of the gp120 core; in other HIV-1 strains, this mutant is predisposed to assume the CD4-bound conformation [44,49,54–59]. If the unliganded HIV-1_BG505_ Env assumes the gp120 core conformation seen in the soluble gp140 SOSIP.664 crystal structure, the S375W mutant should exhibit phenotypes close to those of the wt HIV-1_BG505_ Env. Mutant phenotypes were assessed for full-length HIV-1_BG505_ Envs or, in cases where higher cell-surface expression was desired, Envs with a truncated gp41 cytoplasmic tail.

**Fig 1:**
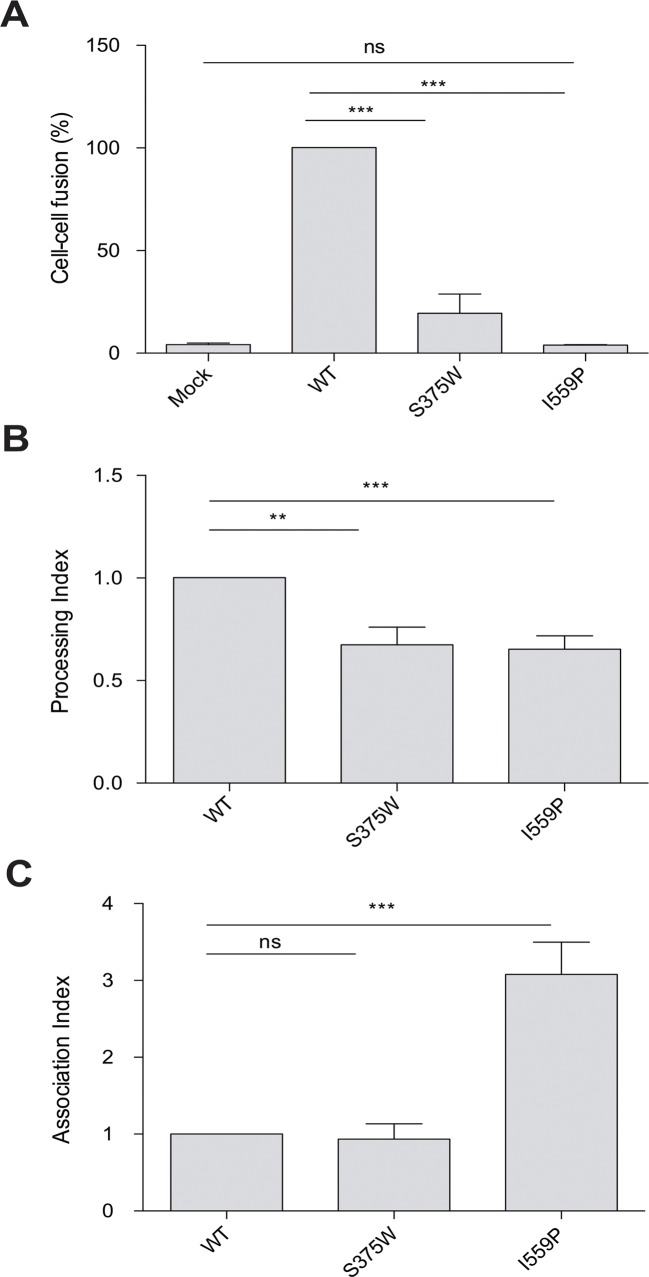
Infectivity, cell-cell fusion, processing and subunit association of Env variants. (**A**). The ability of the indicated HIV-1_BG505_ Env variants to mediate cell-cell fusion was evaluated as described in Materials and Methods. The values shown are normalized to that obtained for wt Env. (**B, C**). Cell lysates and supernatants from ^35^S-labeled cells transiently expressing the indicated HIV-1_BG505_ Env variants were precipitated with a mixture of sera from HIV-1-infected individuals. The precipitated proteins were loaded onto SDS-polyacrylamide gels and analyzed by autoradiography and densitometry to calculate their processing (**B**) and association (**C**) indices, as described in Materials and Methods. Means and SEM derived from at least three independent experiments are shown. The results were compared with a paired t-test, and the degree of significance indicated: ***—p < 0.001, **—p < 0.01, *—p < 0.05 and ns—not significant.

Recombinant luciferase-expressing HIV-1 viruses bearing the full-length wt, I559P or S375W mutant Envs were spinoculated onto Cf2Th cells expressing human CD4 and CCR5 to assess the ability of the Envs to support virus entry [57,59]. The viruses with the wt and S375W Envs infected the CfTh-CD4/CCR5 cells, but the viruses with the I559P Envs did not support infection (data not shown). The I559P Env also failed to mediate the formation of syncytia, whereas the S375W mediated cell-cell fusion, although less efficiently than the wt Env (Fig 1A). The I559P alteration likewise eliminated the ability of an HIV-1_BG505_ Env with a truncated gp41 cytoplasmic tail to mediate virus infection and syncytium formation (Table 1). These results indicate that the I559P change completely compromises the function of the HIV-1_BG505_ Env.

**Table 1:** Phenotypes of HIV-1_BG505_ ΔCT mutants.

Envelope glycoprotein^a^	Residue Location	Processing Index^b^	Association index^b^	Relative infectivity- DTT^c^	Relative infectivity+ DTT^c^	Relative cell-cell fusion - DTT^c^	Relative cell-cell fusion+ DTT^c^
wt		1.00	1.00	1.00	1.00	1.00	1.00
I559P	gp41 HR1	0.66	1.45	<0.01	<0.01	<0.01	<0.01
SOS	A501C/T605C	0.40	1.75	<0.01	0.35	<0.01	0.13
SOSIP	A501C/T605C/I559P	0.43	2.28	<0.01	<0.01	<0.01	<0.01

^a^ A stop codon was introduced in place of the codon for glycine 711 to truncate the cytoplasmic tail (ΔCT) and enhance cell-surface expression of the indicated HIV-1_BG505_ envelope glycoproteins.

^b^ The phenotypes of the wt and mutant Env were determined as described in Materials and Methods.

^c^Infectivity and cell-to-cell fusion levels were normalized to levels obtained with the wt Env in absence (-DTT) or in presence of 1 mM (infectivity) or 2.5 mM (cell-cell fusion) DTT (+DTT). Results represent the mean values derived from at least two independent experiments. Less than 20% deviation from the mean value was typically observed.

The expression, processing and subunit association of the I559P and S375W Env variants were studied by transfection of 293T cells with pcDNA3.1 plasmids expressing the full-length wild-type (wt) and mutant HIV-1_BG505_ Envs, as described [57,58]. The transfected cells were labeled for 5 hours with ^35^S-methionine/cysteine prior to a 16-h chase with medium containing excess methionine and cysteine, as described in Materials and Methods. Cell lysates and supernatants were recovered and precipitated with a mixture of sera from HIV-1-infected individuals. The proteolytic processing of the I559P and S375W Env precursors was modestly decreased, compared with that of the wt Env (Fig 1B). The wt and S375W Envs exhibited similar levels of gp120-gp41 association; the association of the gp120 subunit with the I559P Env trimer was significantly increased, relative to that seen for the wt Env (Fig 1C). Similar phenotypes were observed for an I559P mutant Env with a truncated gp41 cytoplasmic tail (Table 1).

**Fig 2:**
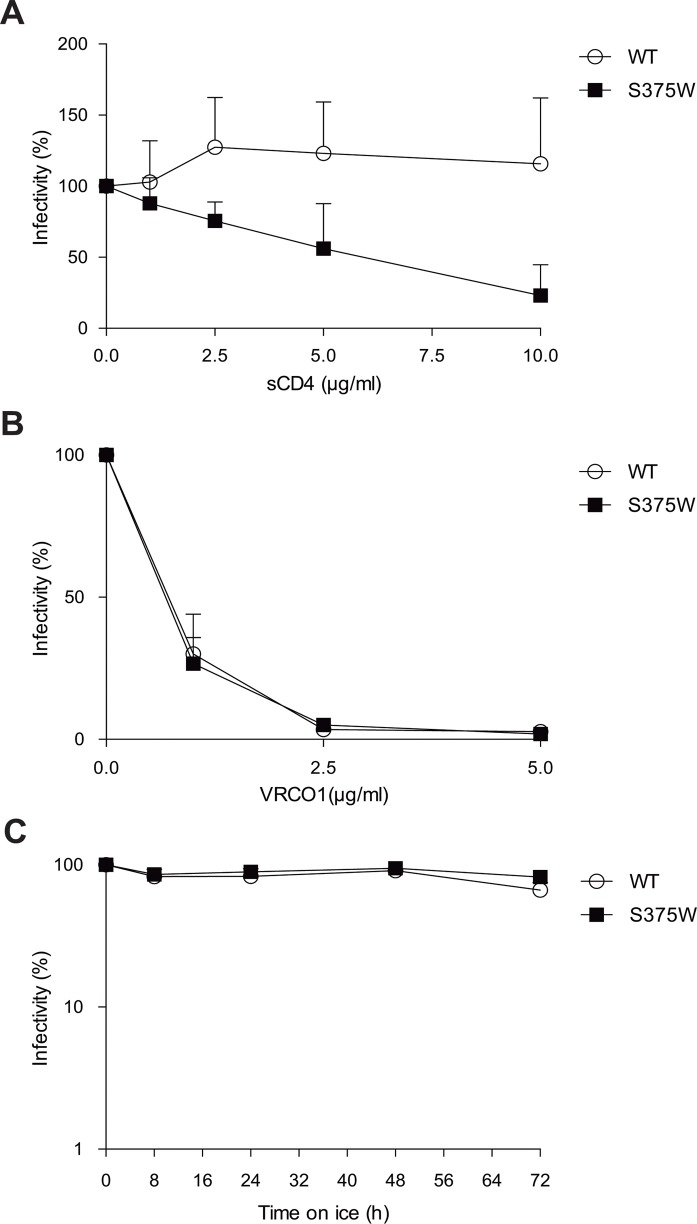
Sensitivity of Env variants to inhibition by sCD4, VRC01 and exposure to cold. Normalized amounts of recombinant luciferase-expressing HIV-1 pseudotyped with the wt and S375W HIV-1_BG505_ Envs were incubated at 37°C with increasing concentrations (0–200 nM) of sCD4 (**A**) or VRC01 (**B**) for 1 hr prior to addition of Cf2Th-CD4/CCR5 cells. In (**C**), recombinant HIV-1 bearing wt or S375W Envs was incubated on ice for different amounts of time [89]. At the indicated time points, aliquots were removed and frozen at -80°C. After completion of the longest incubation, all samples were thawed and infectivity on Cf2Th-CD4/CCR5 cells was measured. Data are representative of results obtained from at least three independent experiments, performed in quadruplicate.

The sensitivity of the wt and S375W viruses to neutralization by soluble CD4 (sCD4) and by the VRC01 and 17b monoclonal antibodies (MAbs) was examined. The S375W virus was more sensitive than the wt virus to sCD4, whereas both viruses were neutralized equivalently by VRC01 (Fig 2A and 2B). Neither virus was sensitive to neutralization by the 17b MAb or to cold inactivation (Fig 2C and data not shown) [56].

### Effect of I559P on the capacity of the SOS variant to mediate membrane fusion under reducing conditions

**Fig 3:**
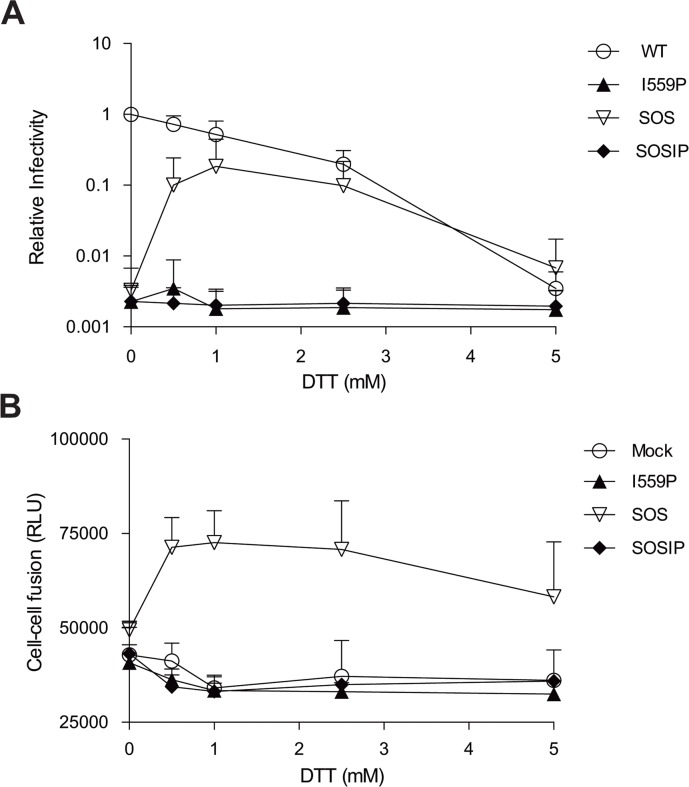
Effect of a reducing agent on the ability of Env variants to mediate infection and cell-cell fusion. (**A**). Normalized amounts of recombinant luciferase-expressing HIV-1 pseudotyped with the wt and mutant HIV-1_BG505_ ΔCT Envs were spin-inoculated onto Cf2Th-CD4/CCR5 cells before adding increasing concentrations of DTT (0–5 mM). (**B**). The ability of the indicated Env variants to mediate cell-cell fusion in the presence of increasing concentrations of DTT was evaluated. The data shown are from a single experiment and are representative of results from at least three independent experiments, performed in quadruplicate.

We evaluated the effect of the I559P change on HIV-1_BG505_ Env function in the absence and presence of the SOS modification. Recombinant luciferase-expressing HIV-1 viruses bearing wt and mutant Envs were spinoculated onto Cf2Th cells expressing human CD4 and CCR5 to assess the ability of the Envs to support virus entry [57,59]. The viruses with the wt and S375W Envs infected the Cf2Th-CD4/CCR5 cells, but the viruses with the I559P, SOS and SOSIP Envs did not infect the cells (Fig 3A and data not shown). Likewise, the I559P, SOS and SOSIP Envs did not detectably mediate cell-cell fusion, whereas the wt and S375W Envs efficiently promoted syncytium formation (Figs 1A, 3B and Table 1). The I559P change significantly compromises the function of the HIV-1_BG505_ Env, and this infectivity defect is not compensated by the SOS cysteine substitutions.

Reduction of the 501–605 disulfide bond has been reported to restore the function of the SOS variant [52,53]. Therefore, we evaluated the infectivity and syncytium-forming ability of our panel of HIV-1_BG505_ Env variants in the presence of increasing concentrations of the reducing agent DTT. As shown in Fig 3A, addition of DTT decreased the infectivity of viral particles bearing the wt Env but, as expected [52,53], enhanced the infectivity (Fig 3A and Table 1) and syncytium-forming ability (Fig 3B and Table 1) of the SOS variant. In contrast, regardless of the concentration of DTT in the medium, the I559P and SOSIP mutants failed to support virus entry or cell-cell fusion. Thus, the I559P change dramatically compromises Env-mediated membrane fusion, in the context of either a wt or SOS Env background.

### Ability of Env variants to achieve the CD4-bound state

**Fig 4:**
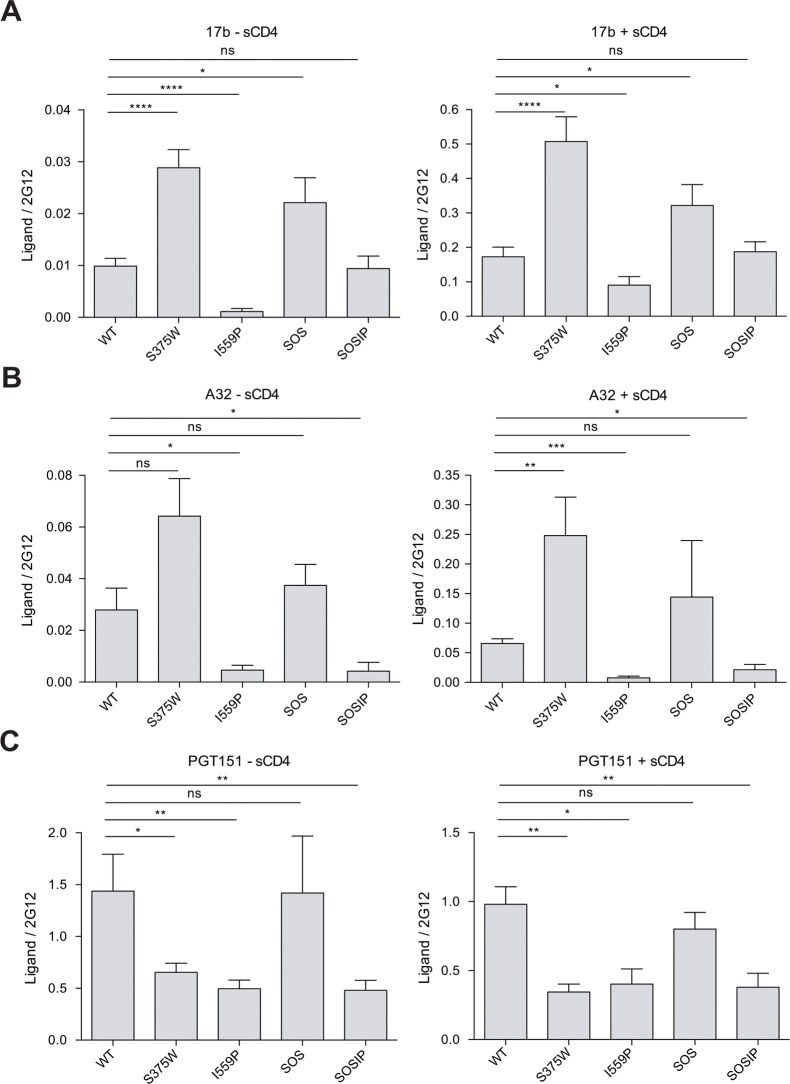
Effect of soluble CD4 on MAb binding to HIV-1_BG505_ Env variants. The binding of 17b (**A**), A32 (**B**) and PGT151 (**C**) MAbs to the indicated HIV-1_BG505_ ΔCT Env variants expressed on the cell surface, in the absence and presence of 80 nM sCD4, is shown. The cell-surface expression of the four Env mutants, as determined by the binding of the 2G12 antibody, was comparable and varied no more than 20% from the level of the wt Env. Each signal was normalized for the relative level of Env expression, using the binding of the 2G12 antibody. Means and SEM derived from at least five independent experiments performed in quadruplicate are shown. Statistical significance is indicated, as described in the Fig 1 legend.

**Fig 5:**
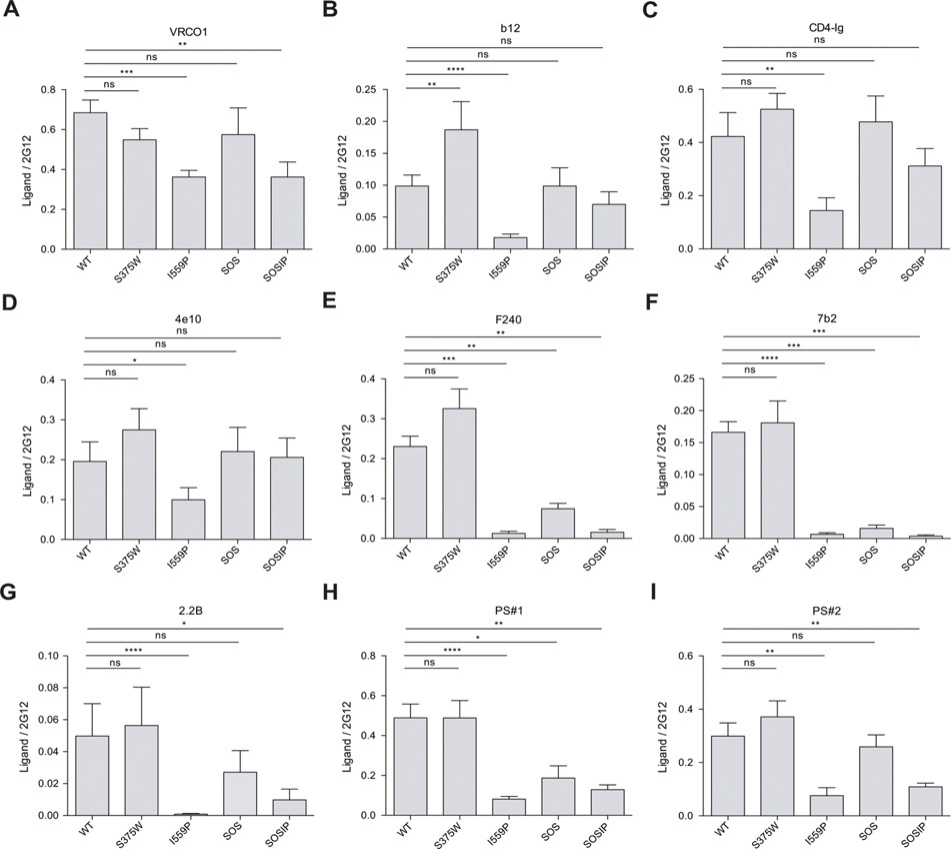
Binding of MAbs and sera from HIV-1-infected individuals to Env variants. The binding of VRC01 (**A**), b12 (**B**), CD4-Ig (**C**), 4e10 (**D**), F240 (**E**), 7b2 (**F**), 2.2B (**G**) or sera from HIV-1-infected individuals (patient sera, PS#1) (**H**) and PS#2 (**I**) to HIV-1_BG505_ ΔCT Env variants expressed on the cell surface is shown. Means and SEM derived from at least five independent experiments performed in quadruplicate are shown. Statistical significance is indicated, as described in the Fig 1 legend.

The recognition of the HIV-1_BG505_ Env variants (wt, S375W, I559P, SOS and SOSIP) by monoclonal antibodies (MAbs) and other ligands was measured by cell-surface ELISA [49,50]. Envs with truncations of the gp41 cytoplasmic tail were used to increase the level of expression on the cell surface. All of the Env variants were expressed on the surface of transfected cells, as determined by binding of the 2G12 MAb, which recognizes a carbohydrate epitope on the gp120 outer domain [60–63] (see Fig 4 and Table 2 legends). The recognition of the Env variants by other Env ligands was normalized to that observed for the 2G12 MAb. The S375W and SOS Envs bound CD4-Ig efficiently, but the binding of CD4-Ig to the I559P mutant was relatively decreased (Fig 5 and Table 2). The spontaneous and sCD4-induced binding of the 17b and A32 MAbs, which recognize distinct CD4-induced (CD4i) gp120 epitopes [59,64,65], to the S375W mutant was increased, relative to that observed for wt Env (Fig 4 and Table 2). Consistent with the results obtained with Envs from other HIV-1 strains [49,54,56–58], filling the Phe43 cavity with a tryptophan residue apparently allows more efficient sampling of the CD4-bound conformation by the HIV-1_BG505_ gp120 core.

**Table 2:** Characterization of ligand binding to selected HIV-1_BG505_ variants by cell-based ELISA.^a^

			HIV-1_BG505_ Env variant				
Env subunit	Epitope	Ligand	wt	S375W	I559P	SOS	SOSIP
gp120	**CD4-BS**	**VRC01**	1.00	0.79	**0.51**	0.82	**0.52**
	**CD4-BS**	**b12**	1.00	**1.72**	**0.19**	1.09	0.73
	**CD4-BS**	**CD4-Ig**	1.00	1.42	**0.31**	1.35	0.74
	**V3**	**19b**	1.00	**1.26**	**0.30**	**1.87**	**0.66**
	**V3**	**GE2 JG8**	1.00	**1.54**	**0.38**	**2.57**	0.96
	**V3 glycans**	**PGT121**	1.00	1.29	**1.73**	1.04	**1.59**
	**V2 glycans**	**PG9 -sCD4**	1.00	0.84	**0.71**	1.01	**0.66**
	**V2 glycans**	**PG9 +sCD4**	0.68	**0.48**	0.61	0.65	0.57
	**CD4i**	**17b -sCD4**	1.00	**3.56**	**<0.01**	**2.68**	1.13
	**CD4i**	**17b +sCD4**	21.80	**61.87**	**12.66**	**35.86**	20.45
	**CD4i**	**A32 -sCD4**	1.00	2.54	**0.03**	1.47	**0.08**
	**CD4i**	**A32 +sCD4**	2.59	**9.81**	**0.19**	5.70	**0.62**
gp120-gp41	**gp120-gp41 interface**	**PGT151 - sCD4**	1.00	**0.45**	**0.34**	0.98	**0.33**
	**gp120-gp41 interface**	**PGT151 + sCD4**	0.68	**0.24**	**0.28**	0.56	**0.26**
	**gp120-gp41 interface**	**35O22 - sCD4**	1.00	**1.47**	1.48	1.00	**1.72**
	**gp120-gp41 interface**	**35O22 + sCD4**	1.02	0.96	**1.84**	1.15	**2.32**
	**gp120-gp41 interface**	**ps** ^**b**^	1.00	0.99	**0.17**	**0.38**	**0.26**
gp41	**MPER**	**4e10**	1.00	1.40	**0.48**	1.24	0.96
	**Cluster I**	**7b2**	1.00	1.08	**0.04**	**0.09**	**0.02**
	**Cluster I**	**F240**	1.00	1.22	**0.03**	**0.25**	**0.07**
	**Cluster II**	**2.2b**	1.00	1.13	**<0.01**	0.55	**0.18**

^a^ For the cell-based ELISA experiments, a stop codon was introduced in place of the codon for glycine 711 to truncate the cytoplasmic tail (ΔCT) and enhance cell-surface expression of the indicated HIV-1_BG505_ envelope glycoproteins. Ligand binding was measured by cell-based ELISA, as described in the Materials and Methods. Each signal was normalized for the relative level of Env expression by the binding of the 2G12 antibody. The cell-surface expression of the four Env mutants, as determined by 2G12 binding, was comparable and exhibited no greater than 20% variation from the level of the wt Env. The ligand-binding values relative to those associated with the wt Env in the absence of sCD4 are reported.

^b^ The reported values for polyclonal serum (ps) from an HIV-1-infected individual represent the average obtained with serum from one HIV-1-infected individual; however, similar results were obtained with sera from more than six randomly selected HIV-1-infected individuals.

Values presented in this table represent the mean of at least four independent experiments done in quadruplicate, with experimental variation typically not more than 20% of the value reported. In bold are values that are statistically different (p≤0.05) from those of the wt Env, as measured by a paired *t* test.

The spontaneous recognition of the I559P mutant by the 17b and A32 MAbs was dramatically reduced, compared with that of the wt Env (Fig 4 and Table 2). The binding of sCD4 partially restored 17b binding to the I559P Env, but A32 binding to the I559P mutant was significantly decreased compared to wt Env, even in the presence of sCD4. These results suggest that the sampling of the CD4-bound conformation by the gp120 subunit is reduced by the I559P change in gp41.

Spontaneous and sCD4-induced binding of the 17b MAb was greater for the SOS mutant than for the wt Env (Fig 4 and Table 2). Moreover, the SOSIP mutant bound the 17b MAb comparably to wt Env, in both the absence and presence of sCD4. Apparently, the SOS change results in a greater propensity to form and expose the 17b epitope on gp120, which can compensate for the negative effects of the I559P alteration on 17b binding. By contrast, similar to the results with the I559P mutant, A32 binding to SOSIP Env in the absence or presence of sCD4 was significantly lower than that of the wt Env. Thus, some but not all of the inhibitory effects of the I559P gp41 change on gp120 transitions to the CD4-bound state can be compensated by the SOS modification.

### Evaluation of the conformation of Env variants with an antibody panel

**Fig 6:**
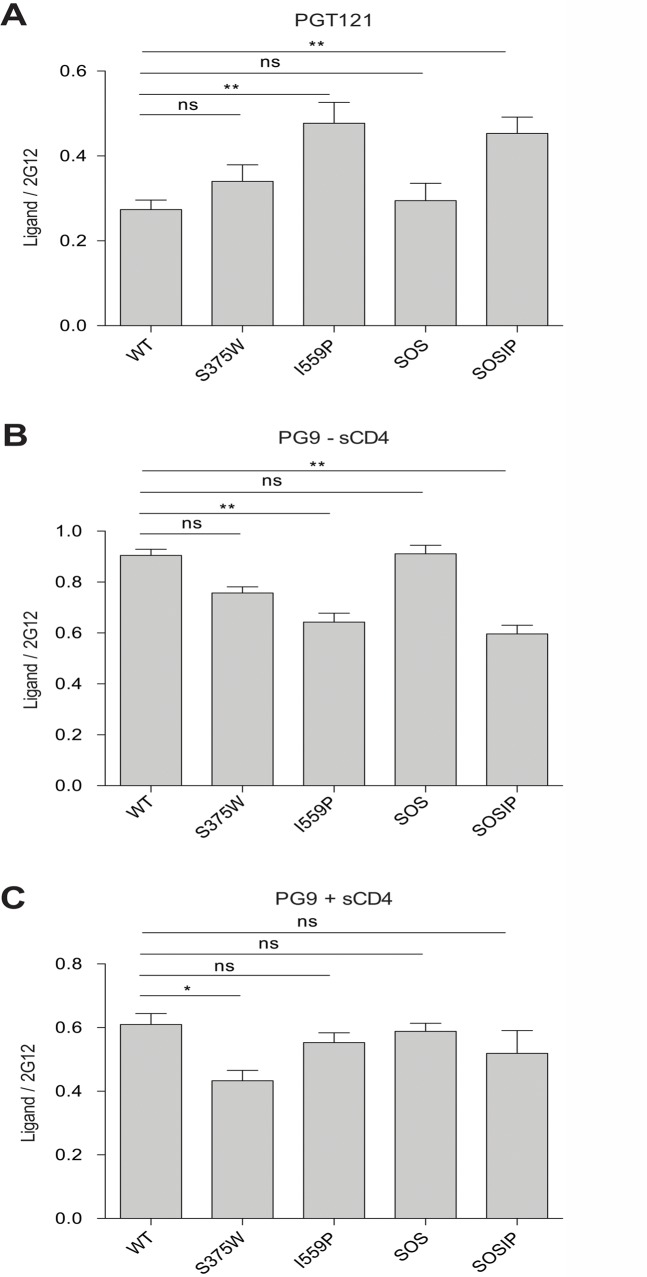
MAb binding to HIV-1_BG505_ Env variants on the cell surface. The binding of PGT121 (**A**) and PG9 (**B** and **C**) to HIV-1_BG505_ Env ΔCT variants expressed on the cell surface was measured by cell-based ELISA. The data in (**C**) were generated in the presence of 80 nM sCD4. The means and SEM derived from at least three independent experiments performed in quadruplicate are shown. Statistical significance was assessed using a paired t-test, and is indicated as described in the legend to Fig 1 of the main text.

Antibody recognition of the cell-surface wt and mutant Envs was evaluated by the cell-based ELISA (Fig 5 and Table 2). The recognition of the I559P Env by the CD4-binding site (CD4BS) MAbs, VRC01 and b12, was decreased relative to that of the wt Env. Decreased binding of b12 to the I559P mutant Env from HIV-1_JR-FL_ has been previously noted [66]. Although b12 binding to the SOSIP Env was better than that of the I559P Env, VRC01 binding to the SOSIP mutant was significantly decreased compared with wt Env. Apparently, the I559P and SOS changes can alter Env quaternary structure so that MAb binding to the CD4-binding site of gp120 is affected. Consistent with this explanation, recognition of the I559P and SOSIP Envs by the PG9 MAb, which recognizes gp120 V2 region glycans in a manner that is sensitive to quaternary trimer conformation [67], was slightly lower than that of the wt Env (Table 2 and Fig 6).

**Fig 7:**
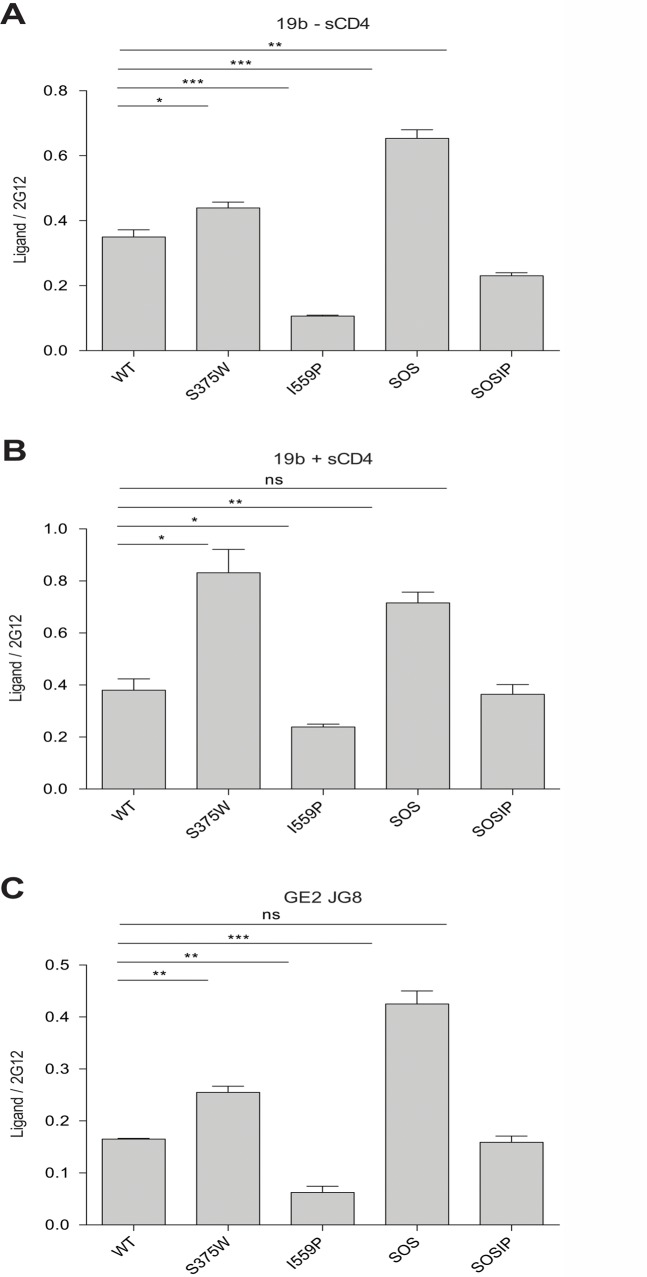
Anti-V3 MAb binding to HIV-1_BG505_ Env variants on the cell surface. The binding of the indicated MAbs to HIV-1_BG505_ Env ΔCT variants expressed on the cell surface was measured by cell-based ELISA. The means and SEM derived from at least three independent experiments performed in quadruplicate are shown. Statistical significance was assessed using a paired t-test, and is indicated as described in the legend to Fig 1 of the main text.

The PGT121 neutralizing MAb, which recognizes a carbohydrate-dependent epitope in the gp120 V3 region [68,69], bound the I559P and SOSIP mutants better than the wt Env (Table 2). By contrast, the 19b and GE2 JG8 anti-V3 MAbs bound the I559P variant less efficiently than the wt Env (Fig 7 and Table 2); similar results were also obtained with the 447 D52 and 2191 anti-V3 MAbs (data not shown). Decreased 19b but not GE2 JG8 binding was also observed for the SOSIP mutant. Both anti-V3 MAbs recognized the SOS mutant more efficiently than the wt Env. These results further suggest that the I559P change in gp41 alters the gp120 conformation in the Env trimer.

The binding of two neutralizing MAbs, PGT151 and 35O22, which recognize gp120-gp41 hybrid epitopes [70–72], to the cell-surface Envs was evaluated in the absence and presence of sCD4 (Table 2). The binding of PGT151 to the S375W, I559P and SOSIP Env mutants was reduced, relative to that of the wt and SOS Envs. Incubation with sCD4 reduced PGT151 binding to all of the Env variants. The 35O22 MAb bound better to the S375W and SOSIP Envs than to the wt Env in the absence of sCD4. In the presence of sCD4, the I559P and SOSIP Envs exhibited a higher-than-wt level of 35O22 MAb binding. These results suggest that the S375W, I559P and SOSIP changes lead to alterations in the relationship between the gp120 and gp41 subunits of the Env trimer.

Additional MAbs directed against gp41 epitopes were tested for recognition of the wt and mutant Envs. MAbs against gp41 Cluster I and Cluster II epitopes exhibited much lower binding to the I559P mutant compared with the wt Env. The relative binding of these MAbs to the SOSIP mutant was also low, indicating that the SOS alteration does not compensate for the I559P change in this instance. In fact, the recognition of the SOS mutant by the Cluster I MAbs was lower than that of the A501C and T605C single-cysteine substitution mutants (data not shown), suggesting that both cysteines may need to form a disulfide bond to cause the observed reduction in recognition of the SOS mutant. A neutralizing MAb directed against the gp41 membrane-proximal external region (MPER), 4E10, exhibited a reduction in recognition of the I559P mutant relative to that of the wt Env. Thus, the I559P change significantly alters the conformation of gp41 in a manner that is minimally compensated by the SOS modification.

We also examined the recognition of the wt and mutant Envs on the cell surface by polyclonal sera from HIV-1-infected individuals. Remarkably, the I559P and SOSIP Envs were recognized less efficiently than the wt and S375W Envs by sera from several HIV-1-infected individuals (Table 2 and Fig 5). Modest decreases in the binding of these sera to the SOS mutant, relative to that of wt Env, were also noted. Thus, the Env complexes with the I559P changes exhibit significant changes in the surface exposure of epitopes recognized by antibodies generated in a subset of HIV-1-infected subjects.

**Fig 8:**
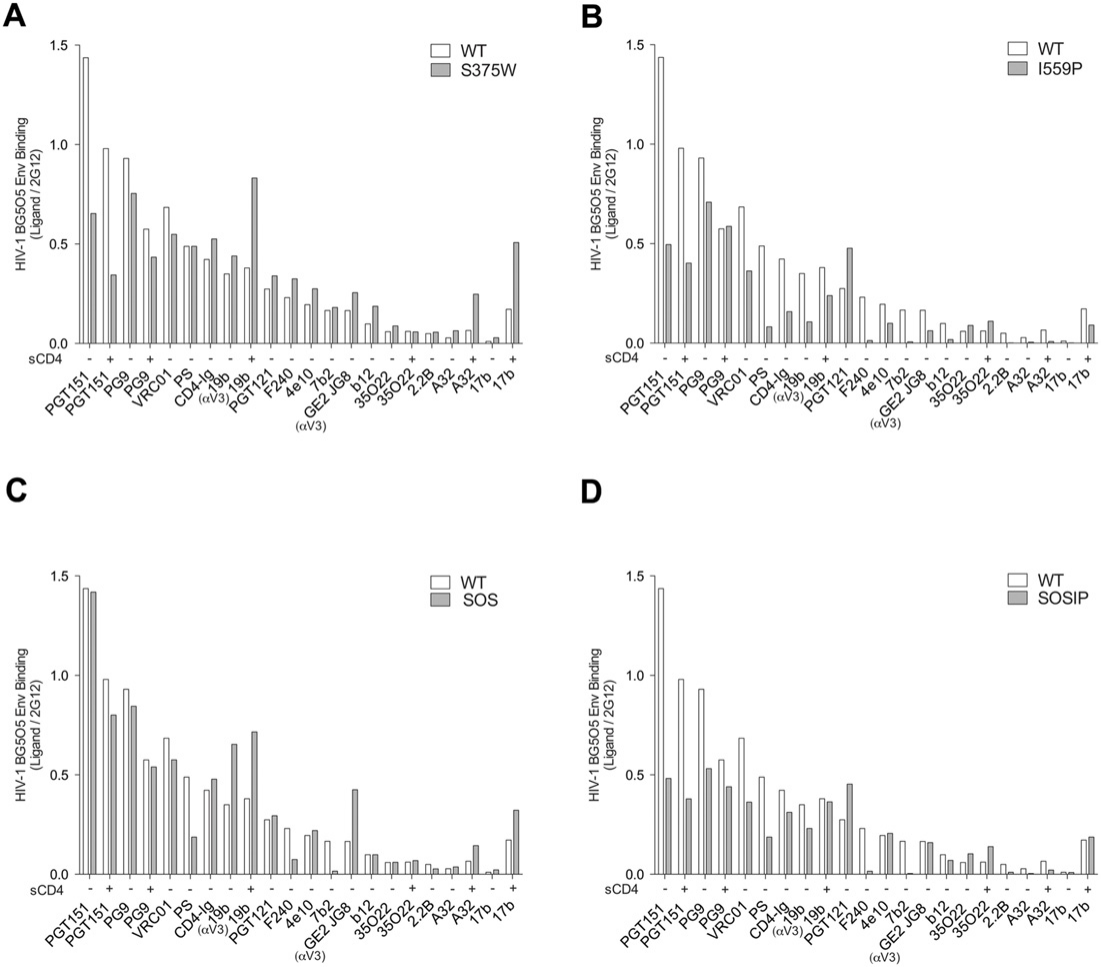
Ligand binding to HIV-1_BG505_ Env variants. The binding of each of the indicated ligands to the HIV-1_BG505_ ΔCT Env variants expressed on the cell surface was measured by the cell-based ELISA, and is plotted relative to the binding of the 2G12 antibody. In some cases, ligand binding was measured in the presence of 80 nM sCD4. The ligands are arranged according to the relative binding to wt Env in the absence of sCD4.

**Fig 9:**
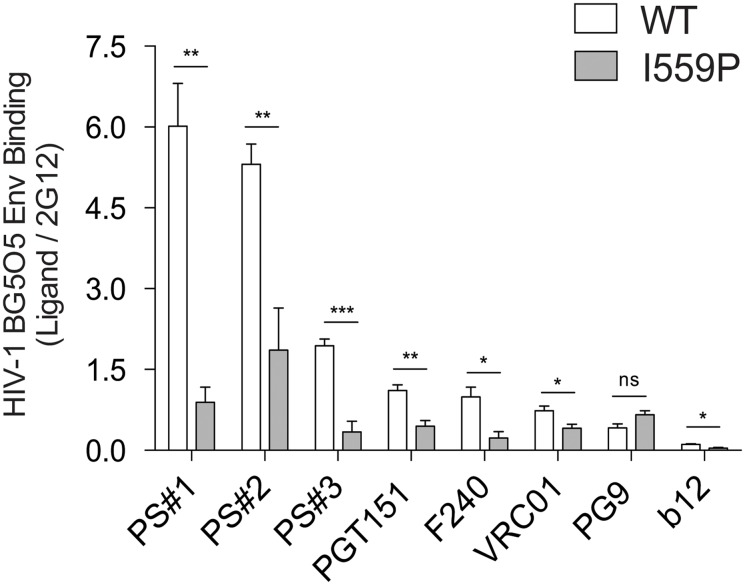
Ligand binding to HIV-1_BG505_ full-length wild-type and I559P Env variants. The binding of wild-type (wt) and I559P HIV-1_BG505_ Env full-length (gp160) variants expressed on the cell surface was measured by cell-based ELISA, and is plotted relative to the binding of the 2G12 antibody. The ligands are arranged according to the relative binding to wt Env. The means and SEM derived from four independent experiments performed in quadruplicate are shown. Statistical significance was assessed using a paired t-test, and the degree of significance is indicated: ***—p < 0.001, **—p < 0.01, *—p < 0.05 and ns—not significant.

Several previous studies have suggested that the long cytoplasmic tail of the HIV-1 Env can influence the structure of the Env ectodomain [42,73–75]. In the cell-based ELISA experiments described above, the Env cytoplasmic tail was truncated to enhance Env expression at the cell surface. To test whether the dramatic effects of the I559P change on Env conformation (Fig 8 and Table 2) were dependent on the truncation of Env, we performed additional cell-based ELISA experiments using the full-length HIV-1_BG505_ gp160 envelope glycoproteins. As shown in Fig 9, the introduction of the I559P change into the full-length gp160 Env closely recapitulated the profound conformational effects described above for Envs lacking the cytoplasmic tail. Indeed, the I559P change resulted in decreased full-length Env recognition by the CD4BS MAbs, VRC01 and b12. Compared with the wt full-length Env, the I559P mutant was recognized less efficiently by the neutralizing MAb, PGT151, by the F240 anti-gp41 MAb, and by sera from HIV-1-infected individuals. Thus, the major conformational changes induced by the I559P change in the membrane-anchored Env are independent of the presence of the gp41 cytoplasmic tail.

## Discussion

The unliganded HIV-1 Env trimer on the viral membrane must retain a high level of potential energy to function as a membrane-fusing machine, and thereby exists in a metastable state [1,76,77]. Whereas binding to the receptors on the target cell membrane triggers Env movement into lower-energy, on-pathway conformations, other influences (amino acid changes, ligand binding, exposure to cold) can lead to more stable Env conformations that are irreversibly inactivated [49,56,76,77]. In creating more stable HIV-1 Env trimers for structural studies, information about the phenotypic consequences of the introduced Env amino acid changes can help to assess whether and how the observed structures fit into the virus entry pathway. Introduction of the helix-breaking proline at residue 559 in gp41 was intended to disrupt the formation of the HR1 coiled coil in the prehairpin intermediate [30]. The I559P change was found to stabilize the formation of trimers in the otherwise heterogeneous soluble gp140 Envs [30,34,35]. All HIV-1 Env trimer preparations currently analyzed by x-ray crystallography contain the I559P change and the SOS alteration that covalently links gp120 and gp41, as well as deletions of part of the gp41 ectodomain, transmembrane region and cytoplasmic tail [37,38]. We investigated the impact of the I559P change, with and without the SOS changes, on the conformation and function of the membrane-anchored Env from HIV-1_BG505_, the same strain of origin as that of the soluble gp140 SOSIP.664 trimer used in most structural studies [36–38,70–72,78–80].

**Fig 10:**
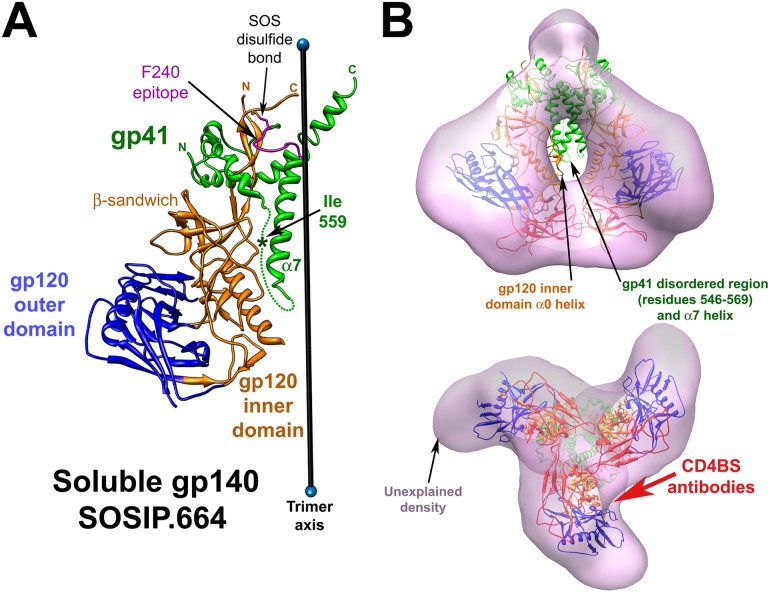
Insights into the I559P phenotypes from the structure of the soluble gp140 SOSIP.664 Env trimer. (**A**). The ribbon structure (PDB 4TVP) of one protomer of the HIV-1_BG505_ soluble gp140 SOSIP.664 Env trimer [38] is shown, oriented with gp41 at the top of the figure. The gp41 subunit is colored green, the gp120 outer domain is colored blue, and the gp120 inner domain and N- and C-termini are colored orange. The approximate location of isoleucine 559 within the disordered gp41 segment (residues 548–568) (dotted line) is indicated by an asterisk. The gp41 α7 helix forms a coiled coil at the trimer axis. The SOS disulfide bond linking gp120 residue 501 and gp41 residue 605 is labeled. The F240 Cluster I gp41 epitope is highlighted in magenta, and is buried near the trimer axis in the soluble gp140 SOSIP.664 structure. The gp120 core is in the CD4-bound conformation, except for the V1/V2 stem, which is not shown. The β-sandwich of the gp120 inner domain, which is involved in the non-covalent association of gp120 and gp41 [90], loosely interacts with the gp41 fusion peptide in this structure. (**B**). The soluble SOSIP.664 Env trimer structure [38] was fitted as a rigid body into the tomogram of the HIV-1 virion Env trimer (grey) (EMD 5019) [40], using UCSF Chimera software [91–93]. The soluble gp140 SOSIP.664 ribbon structure is colored as in **A** and, in addition, the structure of the gp120 V1/V2 and V3 variable regions is shown in red. In the upper panel, the Env spike is oriented such that the viral membrane is at the top of the image. The gp41 disordered region (residues 548–568), including isoleucine 559, and the α7 helix of the soluble gp140 SOSIP.664 structure are out of the virion Env density, as is the α0 helix of the gp120 inner domain. The lower panel shows a view from the perspective of the target cell. Note the density in the gp120 arms of the virion Env tomograms that is not explained by the soluble gp140 SOSIP.664 structure. The approximate angle of approach used by the CD4-binding site (CD4BS) antibodies to engage the HIV-1 Env trimer is indicated by the red arrow. Because these antibodies must access their epitopes by bypassing the adjacent gp120 protomer, their binding is potentially sensitive to changes in the orientation of the gp120 subunits in the quaternary Env trimer structure [42,81].

The phenotypes observed in this study suggest that the I559P mutant differs from wt Env in the conformations of both the gp120 and gp41 subunits. The association of gp120 with the I559P Env trimer was tighter than that observed for wt Env, suggesting that the I559P change alters the relationship among the gp120 and gp41 subunits. Consistent with this, the PGT151 neutralizing MAb, which recognizes a hybrid gp120-gp41 epitope [71,72], bound the I559P Env less efficiently than wt Env. The 35O22 MAb, which recognizes a different gp120-gp41 hybrid epitope [70], bound more efficiently to the I559P mutant than to wt Env in the presence of sCD4. The binding of several ligands (CD4-Ig, VRC01, b12) that recognize a gp120 region near the CD4-binding site was decreased to the I559P mutant, relative to the values seen for wt Env. As these ligands must bypass the adjacent protomer to engage their epitopes [42,81], their binding may be sensitive to differences in quaternary conformation (Fig 10). Recognition of the I559P and SOSIP Envs by the PG9 MAb, whose epitope can be influenced by quaternary Env structure [67], was mildly decreased relative to that of the wt Env. Consistent with an alteration in the gp120-gp41 relationship in the I559P Env, the I559P change in another HIV-1 Env has been reported to result in a loss of sCD4-induced gp120 shedding [66].

The original rationale for the introduction of the I559P change was to destabilize the CD4-bound pre-hairpin intermediate, hopefully predisposing Env to remain in the unliganded state [30]. Indeed, the spontaneous sampling of the CD4-bound conformation by the gp120 subunit appeared to be decreased for the I559P mutant, relative to that of the wt HIV-1_BG505_ Env. In the absence of sCD4, binding of the 17b and A32 MAbs, which prefer the CD4-bound state of gp120 [59,64,65], was reduced to background levels by the I559P change. The I559P change also reduced the binding of several MAbs directed against the gp120 V3 region, another Env element that is exposed better in the CD4-bound state. That an alternation of gp41 influences the conformational states of gp120 suggests an interdependency of the subunits within the Env trimer. For some (17b, GE2 JG8, b12, CD4-Ig) but not all of the gp120-directed ligands, the observed decrease in binding to the I559P Env was compensated by the SOS alteration. It is noteworthy that the SOSIP mutant bound the 17b antibody as well as the wt Env, even in the absence of sCD4. This observation argues against a model in which the I559P and SOS changes lock Env in an unliganded state. Thus, while the I559P mutant resists movement into the CD4-bound state, the I559 change does not guarantee that Env retains an unliganded conformation.

Dramatic reductions in the binding of gp41-directed MAbs were observed for the I559P Env, compared with that of wt Env. The mildly decreased processing of the I559P mutant relative to that of wt Env is expected to increase the exposure of the Cluster I and Cluster II gp41 epitopes [82], so the reduced binding of MAbs to these epitopes is all the more striking. With the exception of the mild decrease in recognition by the 4E10 MAb, the relative decrease in anti-gp41 MAb binding to the I559P Env was not compensated by the SOS change.

Compared with the wt HIV-1_BG505_ Env, the I559P Env mutant was recognized inefficiently by the polyclonal sera from several HIV-1-infected individuals. These sera are known to contain antibodies against multiple epitopes on both gp120 and gp41. These observations support the conclusion that the I559P change causes significant conformational changes in the gp120 and gp41 subunits within the Env trimer. Importantly, the I559P change induced profound conformational changes within both cytoplasmic-tail truncated (Fig 8) and full-length (Fig 9) Envs. Thus, the I559P modification alters Env structure in a manner that is independent of the presence of the cytoplasmic tail, some changes in which have been previously shown to affect the structural and functional properties of the Env ectodomain [42,73–75].

The relative efficiency of binding of each Env ligand to the wt and mutant HIV-1_BG505_ Envs is shown in Fig 8. With a few exceptions (PGT121 and 35O22), the I559P change was associated with decreased recognition by the Env ligands. Importantly, decreased binding of the potent neutralizing antibodies VRC01 and PGT151 is incompatible with a model in which I559P stabilizes the unliganded state of Env. As the broad and potent neutralizing antibodies generally bind more efficiently than non-neutralizing antibodies to the wt Env [76], the epitopes for the potent neutralizing antibodies tended to be preserved on the I559P-containing Envs. In agreement with our data on the membrane SOSIP Env, SOSIP-stabilized soluble gp140 Env trimers retain multiple epitopes for potent, broadly neutralizing antibodies, but are recognized less efficiently by weakly neutralizing or non-neutralizing antibodies [34]. This pattern of epitope exposure could be a desirable quality in an immunogen aimed at eliciting neutralizing antibodies. However, because many potent and broad neutralizing antibodies, particularly those recognizing glycans, tolerate variation in their Env epitopes [61,83–85], they may be less sensitive indicators of conformational disruption than weakly neutralizing antibodies. Therefore, retention of the epitopes for broadly neutralizing antibodies cannot be equated with maintenance of a native functional Env state.

How might the I559P change alter the conformation of the membrane-anchored HIV-1_BG505_ Env? In the crystal structure of the HIV-1_BG505_ soluble gp140 SOSIP.664 trimer, the gp41 region surrounding isoleucine 559 (residues 548–568) is disordered [38] (Fig 10A). As this gp41 region is highly conserved in HIV-1 and is critical for mediating the non-covalent association with gp120 [86], this disorder is unexpected and unlikely to exist in the native unliganded Env (see below). Although Env modifications besides I559P may have contributed to the structure of the soluble gp140 SOSIP.664 glycoprotein observed in the crystal, the isoleucine 559 change is the only alteration within the disordered gp41 segment and thus is a reasonable candidate for the source of this disorder. It is possible that the I559P change results in the disruption of the native gp120-gp41 interface in the context of the membrane-anchored Env and could account for the diverse effects of the I559P change on gp120 and gp41 conformation observed in this and other studies [66].

The I559P change completely eliminated the ability of the primary HIV-1_BG505_ Env to mediate syncytium formation and virus entry, as has been previously observed in the context of a laboratory-adapted HIV-1 Env [87]. Moreover, although the infectivity of the SOS mutant could be rescued by exposure to the reducing agent DTT, the SOSIP variant containing the I559P change was non-functional regardless of the redox environment. Thus, there currently is no evidence that an HIV-1 Env with an I559P change can mediate membrane fusion or support virus entry. At a minimum, the helix-disrupting I559P substitution should impede the formation of the gp41 HR coiled coil [30]. As the antigenic conformation of the I559P Env is not consistent with that expected for the unliganded state of Env (see above), the I559P change may exert other effects that diminish Env function. The relationship between structures of Envs with the I559P alteration and conformations of the functional HIV-1 Env spike remains to be determined.

How might the I559P change stabilize soluble gp140 SOS trimers? With the goal of stabilizing an unliganded state of the soluble gp140 Env trimers, the I559P alteration was intended to disrupt the gp41 HR1 helix and thereby destabilize the downstream pre-hairpin intermediate [30]. The success of this strategy in creating more stable soluble trimers in the unliganded conformation relies on two untested assumptions: 1) that the trimeric associations in the unliganded state are tighter than those in the pre-hairpin intermediate, and 2) that the I559P change does not disrupt the unliganded Env conformation more than that of the pre-hairpin intermediate. The extensive scope of the conformational consequences of the I559P change documented in our study calls into question the latter assumption. This raises the possibility that I559P stabilizes the soluble gp140 SOS trimers by a means other than locking Env in the unliganded state. This possibility is supported by the examination of the crystal structure of the soluble gp140 SOSIP.664 trimer [38] fitted into the tomograms of the native unliganded Env on virions [40] (Fig 10B, upper panel). Two gp41 regions of the soluble gp140 SOSIP.664 trimer are outside of the tomographic density and are therefore incompatible with a native unliganded state of Env. The two gp41 regions are: 1) the long disordered region (residues 548–568) that includes isoleucine 559; and 2) the α7 helix, which forms a trimer-stabilizing coiled coil at the trimer axis (Fig 10A). These two gp41 elements are highly conserved among HIV-1 isolates, so strain variation is unlikely to account for this incompatibility. Instead, we hypothesize that the I559P change disrupts the conformation of the gp41 ectodomain in the soluble gp140 SOSIP.664 trimer and indirectly leads to the formation of the trimer-stabilizing α7 helical coiled coil at the trimer axis. This model resolves the dilemma of explaining stabilization of the soluble gp140 SOSIP.664 trimer by a change in a disordered gp41 region. This model is compatible with two other observations: 1) an α-helical bundle distant from the trimer axis exists in the gp41 ectodomain of an unliganded immature Env trimer [42]; and 2) the substitution of helix-breaking residues (proline and glycine) for isoleucine 559 stabilizes the soluble gp140 trimers better than other amino acid substitutions [30]. Thus, during the folding of the soluble gp140 SOSIP.664 Env, the I559P change might disrupt the native gp41 α-helical bundle, causing the α7 helix and the newly disordered region around isoleucine 559 to collapse towards the trimer axis. This model explains the observed differences between the crystal structure of the soluble gp140 SOSIP.664 trimer [38] and the tomograms of the native unliganded Env spike [40] (Fig 10B, upper panel). The density in the distal arms of the virion Env trimer [40] that is not explained by the soluble gp140 SOSIP.664 structure [37,38] (Fig 10B, lower panel) may reflect the shift of the gp120 subunits towards the trimer axis expected as a consequence of the I559P-induced changes. Indeed, part of the gp120 inner domain in the soluble gp140 SOSIP.664 structure [37,38] abuts the trimer axis and falls outside the density map of the native virion Env spike [40] (Fig 10B, upper panel).

In the crystal structure of the HIV-1_BG505_ soluble gp140 SOSIP.664 trimer, the gp120 core (inner domain, outer domain and bridging sheet) is in the CD4-bound conformation [37,38]. The CD4-bound conformation is a default state assumed by the HIV-1 gp120 core when the constraints operative in the unliganded state are removed [88]; in the soluble gp140 SOSIP.664 structure, the CD4-bound state of the gp120 core may have resulted from a disruption by I559P of the native gp41-mediated constraints on the unliganded gp120 conformation. To investigate the conformation of the gp120 core in the functional HIV-1_BG505_ Env trimer, we introduced a tryptophan residue into the Phe43 cavity, which predisposes the gp120 core to assume the CD4-bound conformation [54,55]. Compared with the wt HIV-1_BG505_ Env, the S375W mutant exhibited greater sensitivity to neutralization by sCD4, but not by the VRC01 CD4BS MAb. The S375W Env bound antibodies like 17b and A32, which prefer the CD4-bound conformation [59,64,65], more efficiently than the wt HIV-1_BG505_ Env, particularly in the presence of sCD4. These results suggest that, like other functional HIV-1 Envs examined to date [40,49,54,56–58,76,77,88], the unliganded HIV-1_BG505_ Env exists in a metastable state with a gp120 core distinct from that found in the CD4-bound state. Thus, the appearance of a CD4-bound gp120 core conformation in the HIV-1_BG505_ soluble gp140 SOSIP.664 structure [37,38] suggests that this structure is unlikely to represent the native unliganded state of Env. Might the soluble gp140 SOSIP.664 structure [37,38] represent another HIV-1 Env conformation on the entry pathway? The soluble gp140 SOSIP.664 structure is clearly incompatible with tomograms of the virion Env spike in its “open,” CD4-bound conformation [40]. A proper assessment of the relationship of the soluble gp140 SOSIP.664 structure to the HIV-1 entry pathway will require additional data. Future efforts to define the structure of the HIV-1 Env trimer in its unliganded and receptor-bound states should recognize and address its natural membrane environment and metastability.
