# Correction: Effects of the I559P gp41 Change on the Conformation and Function of the Human Immunodeficiency Virus (HIV-1) Membrane Envelope Glycoprotein Trimer

**DOI:** 10.1371/journal.pone.0129405

**Published:** 2015-05-26

**Authors:** The PLOS ONE Staff

## Notice of Republication

This article was republished on May 4, 2015, due to figures 8, 9, and 10 being ordered incorrectly. The publisher apologizes for this error. Please download the PDF again to view the corrected article. The originally published, uncorrected article and the republished, corrected article are provided here for reference.

## Supporting Information

S1 File Originally published, uncorrected article (PDF)

S2 File Republished, corrected article (PDF)
