# Supplementary material for: Correction: Effects of the I559P gp41 Change on the Conformation and Function of the Human Immunodeficiency Virus (HIV-1) Membrane Envelope Glycoprotein Trimer
Source: PLoS One. 2015 May 26;10(5):e0129405. doi: 10.1371/journal.pone.0129405 (PMC4444099; doi:10.1371/journal.pone.0129405)
Supplement: S2 File — (PDF) [file pone.0129405.s002.pdf]

RESEARCH ARTICLE

Effects of the I559P gp41 Change on the Conformation and Function of the Human Immunodeficiency Virus (HIV-1) Membrane Envelope Glycoprotein Trimer

Nirmin Alsahafi[1,2☯], **Olfa Debbeche**[2,3☯], **Joseph Sodroski**[4,5,6]*, **Andrés Finzi**[1,2,3]*

1 Department of Microbiology and Immunology, McGill University, Montreal, H3A 2B4 Quebec, Canada, **2** Centre de Recherche du Centre Hospitalier de l'Université de Montréal, Montréal, H2X 0A9, Québec, Canada, **3** Department of Microbiology, Infectiology and Immunology, Université de Montréal, Montréal, H3C 3J7 Quebec, Canada, **4** Department of Cancer Immunology and AIDS, Dana-Farber Cancer Institute, Boston, Massachusetts, 02115, United States of America, **5** Department of Microbiology & Immunobiology, Harvard Medical School, Boston, Massachusetts, 02115, United States of America, **6** Department of Immunology and Infectious Diseases, Harvard School of Public Health, Boston, Massachusetts, 02115, United States of America

☯ These authors contributed equally to this work.
* joseph_sodroski@dfci.harvard.edu (JS); andres.finzi@umontreal.ca (AF)

OPEN ACCESS

Citation: Alsahafi N, Debbeche O, Sodroski J, Finzi A (2015) Effects of the I559P gp41 Change on the Conformation and Function of the Human Immunodeficiency Virus (HIV-1) Membrane Envelope Glycoprotein Trimer. PLoS ONE 10(4): e0122111. doi:10.1371/journal.pone.0122111

Academic Editor: Chen Liang, Lady Davis Institute for Medical Research, CANADA

Received: December 29, 2014

Accepted: February 20, 2015

Published: April 7, 2015

Copyright: © 2015 Alsahafi et al. This is an open access article distributed under the terms of the Creative Commons Attribution License, which permits unrestricted use, distribution, and reproduction in any medium, provided the original author and source are credited.

Data Availability Statement: All relevant data are within the paper.

Funding: This work was supported by grants from the National Institutes of Health (AI24755 and AI100645) and by the late William F. McCarty-Cooper to JS and by a Canada Foundation for Innovation Program Leader grant #29866, by CIHR operating grants #119334 and #134117, by a FRQS Establishment of Young Scientist grant #26702 to AF. AF is the recipient of a Canada Research Chair on Retroviral Entry #950-229930. NA is the recipient of a

Abstract

The mature human immunodeficiency virus (HIV-1) envelope glycoprotein (Env) trimer is produced by proteolytic cleavage of a precursor and consists of three gp120 exterior and three gp41 transmembrane subunits. The metastable Env complex is induced to undergo conformational changes required for virus entry by the binding of gp120 to the receptors, CD4 and CCR5/CXCR4. An isoleucine-to-proline change (I559P) in the gp41 ectodomain has been used to stabilize soluble forms of HIV-1 Env trimers for structural characterization and for use as immunogens. In the native membrane-anchored HIV-1_{BG505} Env, the I559P change modestly decreased proteolytic maturation, increased the non-covalent association of gp120 with the Env trimer, and resulted in an Env conformation distinctly different from that of the wild-type HIV-1_{BG505} Env. Compared with the wild-type Env, the I559P Env was recognized inefficiently by polyclonal sera from HIV-1-infected individuals, by several gp41-directed antibodies, by some antibodies against the CD4-binding site of gp120, and by antibodies that preferentially recognize the CD4-bound Env. Some of the gp120-associated antigenic differences between the wild-type HIV-1_{BG505} Env and the I559P mutant were compensated by the SOS disulfide bond between gp120 and gp41, which has been used to stabilize cleaved soluble Env trimers. Nonetheless, regardless of the presence of the SOS changes, Envs with proline 559 were recognized less efficiently than Envs with isoleucine 559 by the VRC01 neutralizing antibody, which binds the CD4-binding site of gp120, and the PGT151 neutralizing antibody, which binds a hybrid gp120-gp41 epitope. The I559P change completely eliminated the ability of the HIV-1_{BG505} Env to mediate cell-cell fusion and virus entry, and abolished the capacity of the SOS Env to support virus infection in the

King Abdullah scholarship for higher education from the Saudi Government.

Competing Interests: The authors have declared that no competing interests exist.

presence of a reducing agent. These results suggest that differences exist between the quaternary structures of functional Env spikes and I559P Envs.

Introduction

Human immunodeficiency virus (HIV-1) entry into the host cell is mediated by the viral envelope glycoproteins (Envs), which are derived by proteolytic cleavage of a trimeric gp160 Env precursor [1–3]. The resulting mature Env complex is composed of three gp120 exterior subunits and three gp41 transmembrane subunits. The metastable Env trimer undergoes conformational changes upon binding of gp120 to the receptors, CD4 and CCR5/CXCR4, that promote the fusion of the viral and target cell membranes by gp41 [1,4–17]. CD4 binding results in the pre-hairpin intermediate, in which the gp41 heptad repeat 1 (HR1) α-helical coiled coil is formed and exposed [18–22]. The binding of gp120 to either CCR5 or CXCR4 is thought to trigger the formation of a stable gp41 six-helix bundle required for fusion of the viral and target cell membranes [4–7,18–20,22–24].

Metastability and membrane localization are essential for HIV-1 Env function, but create difficulties for the preparation of stable, homogeneous Envs for structural analysis. Various approaches have been used to produce tractable soluble Env trimers lacking the transmembrane anchor and cytoplasmic tail [25–34]. Soluble gp140 Envs typically exhibit multiple oligomeric species in solution, indicating the weakness of the interprotomer contacts in the HIV-1 Env ectodomain and motivating efforts to improve their stability [25–34]. Proteolytically processed versions of soluble gp140 Env have been produced by the introduction of an artificial disulfide bond (SOS) linking the gp120 and gp41 subunits [31–34]. To attempt to destabilize the pre-hairpin intermediate and produce Envs in the mature, unliganded state, the helix-breaking isoleucine-to-proline change (I559P) has been introduced into the gp41 ectodomain [30]. The I559P change was found to stabilize soluble SOS gp140 trimers, and the I559P alteration has been combined with the SOS disulfide bond and deletion of the membrane-proximal gp41 region to produce soluble gp140 SOSIP.664 glycoproteins [34,35]. X-ray and cryoelectron microscopy (cryo-EM) structures of these soluble gp140 SOSIP glycoproteins bound to Fab fragments of neutralizing antibodies have been solved [36–39]. These structures provide information on the nature and arrangement of neutralizing antibody epitopes on soluble HIV-1 Env trimers. However, the soluble gp140 SOSIP.664 structures differ from cryo-EM maps of unliganded, proteolytically mature Env trimers derived from virions or uncleaved Env trimers from cell surfaces [40–43]. Moreover, the structure of the gp120 core (gp120 lacking the V1/V2 and V3 variable regions and N/C termini) in the soluble gp140 SOSIP.664 Env crystals unexpectedly resembles the CD4-bound conformation,[36–38,44]. These observations suggest possible differences in conformation between the soluble gp140 SOSIP.664 trimers and the unliganded functional Env spike on the surface of infected cells and virions. Here we investigate the impact of the I559P change on the conformation and function of the membrane-anchored Env from the primary HIV-1_{BG505} strain, from which the crystallized SOSIP.664 Env trimers [37,38] were derived. We also investigate the conformation of the gp120 core in the HIV-1_{BG505} Env by studying the phenotypes of a mutant, S375W, that predisposes gp120 to assume the CD4-bound state.

Materials and Methods

Cell lines

293T human embryonic kidney, Cf2Th canine thymocytes (American Type Culture Collection), HOS and TZM-bl cell lines (NIH AIDS Research and Reference Reagent Program) were grown at 37°C and 5% CO_2 in Dulbecco's modified Eagle's medium (Invitrogen) containing 10% fetal bovine serum (Sigma) and 100 μg/ml of penicillin-streptomycin (Mediatech). Cf2Th cells stably expressing human CD4 and CCR5 [45] were grown in medium supplemented with 0.4 mg/ml of G418 (Invitrogen) and 0.15 mg/ml of hygromycin B (Roche Diagnostics). The TZM-bl cell line is a HeLa cell line stably expressing high levels of CD4 and CCR5 and possessing an integrated copy of the luciferase gene under control of the HIV-1 long terminal repeat [46].

Site-directed mutagenesis

The sequence of full-length clade A HIV-1_{BG505} Env [47] was codon-optimized (GenScript) and cloned into the expression plasmid pcDNA3.1. Mutations were introduced individually or in combination into this plasmid, pcDNA3.1-BG505. Site-directed mutagenesis was performed using the QuikChange II XL site-directed mutagenesis protocol (Stratagene). For cell-based ELISA experiments, a stop codon was introduced to replace the codon for Gly 711, truncating the cytoplasmic tail (ΔCT) and enhancing cell-surface expression of selected HIV-1_{BG505} Env variants. The presence of the desired mutations was determined by automated DNA sequencing. The numbering of the HIV-1 Env amino acid residues is based on that of the prototypic HXBc2 strain of HIV-1, where position 1 is the initial methionine [48].

Immunoprecipitation of envelope glycoproteins

For pulse-labeling experiments, $3X10^5$ 293T cells were cotransfected by the calcium phosphate method with the pcDNA3.1-BG505 vector expressing wild-type or mutant envelope glycoproteins. One day after transfection, cells were metabolically labeled for 5 h with 100 μCi/mL [^{35}S]methionine-cysteine ([^{35}S] Protein Labeling Mix; Perkin-Elmer) in Dulbecco's modified Eagle's medium lacking methionine and cysteine and supplemented with 5% dialyzed fetal bovine serum and then chased over-night with complete DMEM medium containing excess of methionine and cysteine. Cells were subsequently lysed in RIPA buffer (140 mM NaCl, 8 mM Na_2HPO_4, 2 mM NaH_2PO_4, 1% NP40, 0.05% sodium dodecyl sulfate (SDS)). Precipitation of radiolabeled HIV-1_{BG505} envelope glycoproteins from cell lysates or medium was performed with a mixture of sera from HIV-1-infected individuals for 1 hour at 4°C in the presence of 50 μl of 10% Protein A-Sepharose (American BioSciences). Written informed consent was obtained from all study participants, research adhered to the ethical guidelines of CRCHUM and was reviewed and approved by CRCHUM institutional review board (ethics committee). Sera was collected during Ficoll isolation of PBMCs and conserved at -80°C. Sera aliquots were heat-inactivated for 30 min at 56°C and stored at 4°C until ready to use in subsequent experiments.

Processing and association indices were determined by precipitation of radiolabeled cell lysates and supernatants with mixtures of sera from HIV-1-infected individuals. The association index is a measure of the ability of the mutant gp120 molecule to remain associated with the Env trimer complex on the expressing cell, relative to that of the wild-type Env trimers. The association index is calculated as follows: association index = ([mutant gp120]$_{cell}$ × [wild-type gp120]$_{supernatant}$)/ ([mutant gp120]$_{supernatant}$ × [wild-type gp120]$_{cell}$). The processing index is a measure of the conversion of the mutant gp160 Env precursor to mature gp120, relative to that

of the wild-type Env trimers. The processing index was calculated by the formula: processing index = ([total gp120]$_{mutant}$ × [gp160]$_{wild\text{-}type}$)/ ([gp160]$_{mutant}$ × [total gp120]$_{wild\text{-}type}$).

Cell-based ELISA

Detection of trimeric Env on the surface of HOS cells was performed by cell-based ELISA, as described [49,50]. Briefly, HOS cells were seeded in 96-well plates (2x10^4 cells per well) and transfected the next day with 150 ng of ΔCT or 250 ng of full-length (gp160) Env-expressing plasmids using the standard polyethylenimine (PEI, Polyscience Inc, PA, USA) transfection method. Two days later, cells were washed twice with blocking buffer (10 mg/ml non-fat dry milk, 1.8 mM $CaCl_2$, 1 mM $MgCl_2$, 25 mM Tris,pH 7.5 and 140 mM NaCl) and then incubated for 1 h at room temperature with 20 nM CD4-Ig or anti-HIV-1 Env monoclonal antibodies recognizing CD4-induced gp120 epitopes (17b, A32), CD4-binding site (CD4BS) gp120 epitopes (VRC01, b12), gp120 V2 glycans (PG9), the gp120 V3 region (19b, GE2 JG8), gp120 V3 glycans (PGT121), gp120 outer domain glycans (2G12), the gp120-gp41 interface (PGT151, 35O22), and gp41 Cluster I (F240, 7B.2), Cluster II (2.2B) and MPER (4e10) epitopes. When indicated, cells were pre-incubated for 40 min at room temperature in absence or presence of 80 nM sCD4 before adding the first antibody. All ligands were diluted in blocking buffer. A horseradish peroxidase-conjugated antibody specific for the Fc region of human IgG (Pierce) was then incubated with the samples for 45 minutes at room temperature. For all conditions, cells were washed 5 times with blocking buffer and 5 times with washing buffer. HRP enzyme activity was determined after the addition of 30 μl per well of a 1:1 mix of Western Lightning oxidizing and luminol reagents (Perkin Elmer Life Sciences). Light emission was measured with an LB 941 TriStar luminometer (Berthold Technologies).

Single-round luciferase-expressing viruses

Single-round viruses containing the firefly luciferase gene were produced by calcium phosphate transfection of 293T cells with the HIV-1 proviral vector pNL4.3Env$^-$Luc and the pcDNA3.1 BG505 plasmid expressing the wild-type or mutant HIV-1$_{BG505}$ envelope glycoproteins at a ratio of 2:1. Two days after transfection, the cell supernatants were harvested; the reverse transcriptase activities of all viruses were measured as described previously [51]. The virus-containing supernatants were stored in aliquots at -80°C.

Infection by single-round luciferase-expressing viruses

Cf2Th-CD4/CCR5 target cells were seeded at a density of 5 X 10^3 cells/well in 96-well luminometer-compatible tissue culture plates (Corning) 24 h before infection. Normalized amounts of viruses (as evaluated by reverse transcriptase activity of the viral stocks) were then added to the target cells followed by spin infection at 800 g for 1 h in 96-well plates at 25°C. When indicated, several concentrations (0.5, 1, 2.5 and 5 mM) of dithiothreitol (DTT) were added to the cells after the spin infection. In all cases, cells were incubated after the spin infection for 48 h at 37°C; the medium was then removed from each well, and the cells were lysed by the addition of 30 μl of passive lysis buffer (Promega) and three freeze-thaw cycles. An LB 941 TriStar luminometer (Berthold Technologies) was used to measure the luciferase activity of each well after the addition of 100 μl of luciferin buffer (15 mM $MgSO_4$, 15 mM KPO_4 [pH 7.8], 1 mM ATP, and 1 mM dithiothreitol) and 50 μl of 1 mM D-luciferin potassium salt (Prolume).

Cell-cell fusion

To assess cell-to-cell fusion, 3 X 10^5 293T cells were cotransfected by the calcium phosphate method with an HIV-1 Tat-expressing plasmid, pLTR-Tat, and the pcDNA3.1 BG505 vector expressing the HIV-1_{BG505} envelope glycoprotein variants. Sixteen hours later, the cells were washed with fresh DMEM. Two days after transfection, the 293T cells were removed from the plate and counted. Then 3 X 10^4 293T cells were added to TZM-bl target cells that were seeded at a density of 2 X 10^4 cells/well in 96-well luminometer-compatible tissue culture plates (Corning) 24 hours before the assay. When indicated, several concentrations (0.5, 1, 2.5 and 5 mM) of DTT were added to the cells after 30 min of co-incubation at room temperature and before incubation at 37°C. After six hours at 37°C, cells were lysed by the addition of 30 μl passive lysis buffer (Promega) and three freeze-thaw cycles. Luciferase activity in each well was measured as described above.

Results

Effect of the I559P and S375W changes on the function, processing and subunit association of the HIV-1_{BG505} Envs

In this study, the phenotypes of the I559P mutant were evaluated in the background of the wild-type (wt) HIV-1_{BG505} Env and in an Env with the SOS cysteine substitutions [52,53]. Because the structure of the gp120 core in the soluble gp140 SOSIP.664 Env crystals unexpectedly resembles the CD4-bound conformation [36–38,44], we also studied the S375W mutant, in which the aromatic ring of a tryptophan residue fills the Phe43 cavity of the gp120 core; in other HIV-1 strains, this mutant is predisposed to assume the CD4-bound conformation [44,49,54–59]. If the unliganded HIV-1_{BG505} Env assumes the gp120 core conformation seen in the soluble gp140 SOSIP.664 crystal structure, the S375W mutant should exhibit phenotypes close to those of the wt HIV-1_{BG505} Env. Mutant phenotypes were assessed for full-length HIV-1_{BG505} Envs or, in cases where higher cell-surface expression was desired, Envs with a truncated gp41 cytoplasmic tail.

Recombinant luciferase-expressing HIV-1 viruses bearing the full-length wt, I559P or S375W mutant Envs were spinoculated onto Cf2Th cells expressing human CD4 and CCR5 to assess the ability of the Envs to support virus entry [57,59]. The viruses with the wt and S375W Envs infected the CfTh-CD4/CCR5 cells, but the viruses with the I559P Envs did not support infection (data not shown). The I559P Env also failed to mediate the formation of syncytia, whereas the S375W mediated cell-cell fusion, although less efficiently than the wt Env (Fig 1A). The I559P alteration likewise eliminated the ability of an HIV-1_{BG505} Env with a truncated gp41 cytoplasmic tail to mediate virus infection and syncytium formation (Table 1). These results indicate that the I559P change completely compromises the function of the HIV-1_{BG505} Env.

The expression, processing and subunit association of the I559P and S375W Env variants were studied by transfection of 293T cells with pcDNA3.1 plasmids expressing the full-length wild-type (wt) and mutant HIV-1_{BG505} Envs, as described [57,58]. The transfected cells were labeled for 5 hours with ^{35}S-methionine/cysteine prior to a 16-h chase with medium containing excess methionine and cysteine, as described in Materials and Methods. Cell lysates and supernatants were recovered and precipitated with a mixture of sera from HIV-1-infected individuals. The proteolytic processing of the I559P and S375W Env precursors was modestly decreased, compared with that of the wt Env (Fig 1B). The wt and S375W Envs exhibited similar levels of gp120-gp41 association; the association of the gp120 subunit with the I559P Env trimer was significantly increased, relative to that seen for the wt Env (Fig 1C). Similar phenotypes were observed for an I559P mutant Env with a truncated gp41 cytoplasmic tail (Table 1).

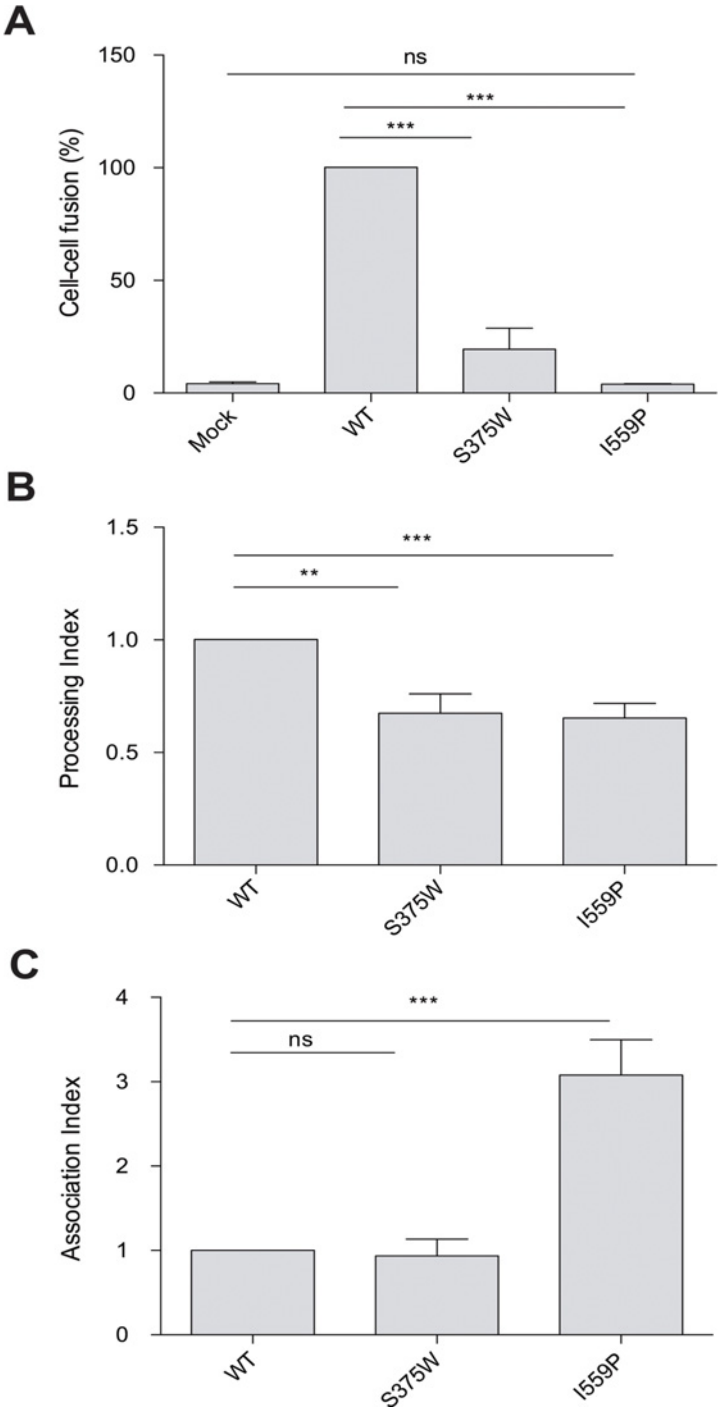

Fig 1. Infectivity, cell-cell fusion, processing and subunit association of Env variants. (**A**). The ability of the indicated HIV-1_{BG505} Env variants to mediate cell-cell fusion was evaluated as described in Materials and Methods. The values shown are normalized to that obtained for wt Env. (**B, C**). Cell lysates and supernatants from ^{35}S-labeled cells transiently expressing the indicated HIV-1_{BG505} Env variants were precipitated with a mixture of sera from HIV-1-infected individuals. The precipitated proteins were loaded onto SDS-polyacrylamide gels and analyzed by autoradiography and densitometry to calculate their processing (**B**) and association (**C**) indices, as described in Materials and Methods. Means and SEM derived from at least three independent experiments are shown. The results were compared with a paired t-test, and the degree of significance indicated: ***—$p < 0.001$, **—$p < 0.01$, *—$p < 0.05$ and ns—not significant.

doi:10.1371/journal.pone.0122111.g001

Table 1. Phenotypes of HIV-1$_{BG505}$ ΔCT mutants.

Envelope glycoprotein[a]	Residue Location	Processing Index[b]	Association index[b]	Relative infectivity- DTT[c]	Relative infectivity+ DTT[c]	Relative cell-cell fusion - DTT[c]	Relative cell-cell fusion+ DTT[c]
wt		1.00	1.00	1.00	1.00	1.00	1.00
I559P	gp41 HR1	0.66	1.45	<0.01	<0.01	<0.01	<0.01
SOS	A501C/T605C	0.40	1.75	<0.01	0.35	<0.01	0.13
SOSIP	A501C/T605C/ I559P	0.43	2.28	<0.01	<0.01	<0.01	<0.01

[a] A stop codon was introduced in place of the codon for glycine 711 to truncate the cytoplasmic tail (ΔCT) and enhance cell-surface expression of the indicated HIV-1$_{BG505}$ envelope glycoproteins.

[b] The phenotypes of the wt and mutant Env were determined as described in Materials and Methods.

[c]Infectivity and cell-to-cell fusion levels were normalized to levels obtained with the wt Env in absence (-DTT) or in presence of 1 mM (infectivity) or 2.5 mM (cell-cell fusion) DTT (+DTT). Results represent the mean values derived from at least two independent experiments. Less than 20% deviation from the mean value was typically observed.

doi:10.1371/journal.pone.0122111.t001

The sensitivity of the wt and S375W viruses to neutralization by soluble CD4 (sCD4) and by the VRC01 and 17b monoclonal antibodies (MAbs) was examined. The S375W virus was more sensitive than the wt virus to sCD4, whereas both viruses were neutralized equivalently by VRC01 (Fig 2A and 2B). Neither virus was sensitive to neutralization by the 17b MAb or to cold inactivation (Fig 2C and data not shown) [56].

Effect of I559P on the capacity of the SOS variant to mediate membrane fusion under reducing conditions

We evaluated the effect of the I559P change on HIV-1$_{BG505}$ Env function in the absence and presence of the SOS modification. Recombinant luciferase-expressing HIV-1 viruses bearing wt and mutant Envs were spinoculated onto Cf2Th cells expressing human CD4 and CCR5 to assess the ability of the Envs to support virus entry [57,59]. The viruses with the wt and S375W Envs infected the Cf2Th-CD4/CCR5 cells, but the viruses with the I559P, SOS and SOSIP Envs did not infect the cells (Fig 3A and data not shown). Likewise, the I559P, SOS and SOSIP Envs did not detectably mediate cell-cell fusion, whereas the wt and S375W Envs efficiently promoted syncytium formation (Figs 1A, 3B and Table 1). The I559P change significantly compromises the function of the HIV-1$_{BG505}$ Env, and this infectivity defect is not compensated by the SOS cysteine substitutions.

Reduction of the 501–605 disulfide bond has been reported to restore the function of the SOS variant [52,53]. Therefore, we evaluated the infectivity and syncytium-forming ability of our panel of HIV-1$_{BG505}$ Env variants in the presence of increasing concentrations of the reducing agent DTT. As shown in Fig 3A, addition of DTT decreased the infectivity of viral particles bearing the wt Env but, as expected [52,53], enhanced the infectivity (Fig 3A and Table 1) and syncytium-forming ability (Fig 3B and Table 1) of the SOS variant. In contrast, regardless of the concentration of DTT in the medium, the I559P and SOSIP mutants failed to support virus entry or cell-cell fusion. Thus, the I559P change dramatically compromises Env-mediated membrane fusion, in the context of either a wt or SOS Env background.

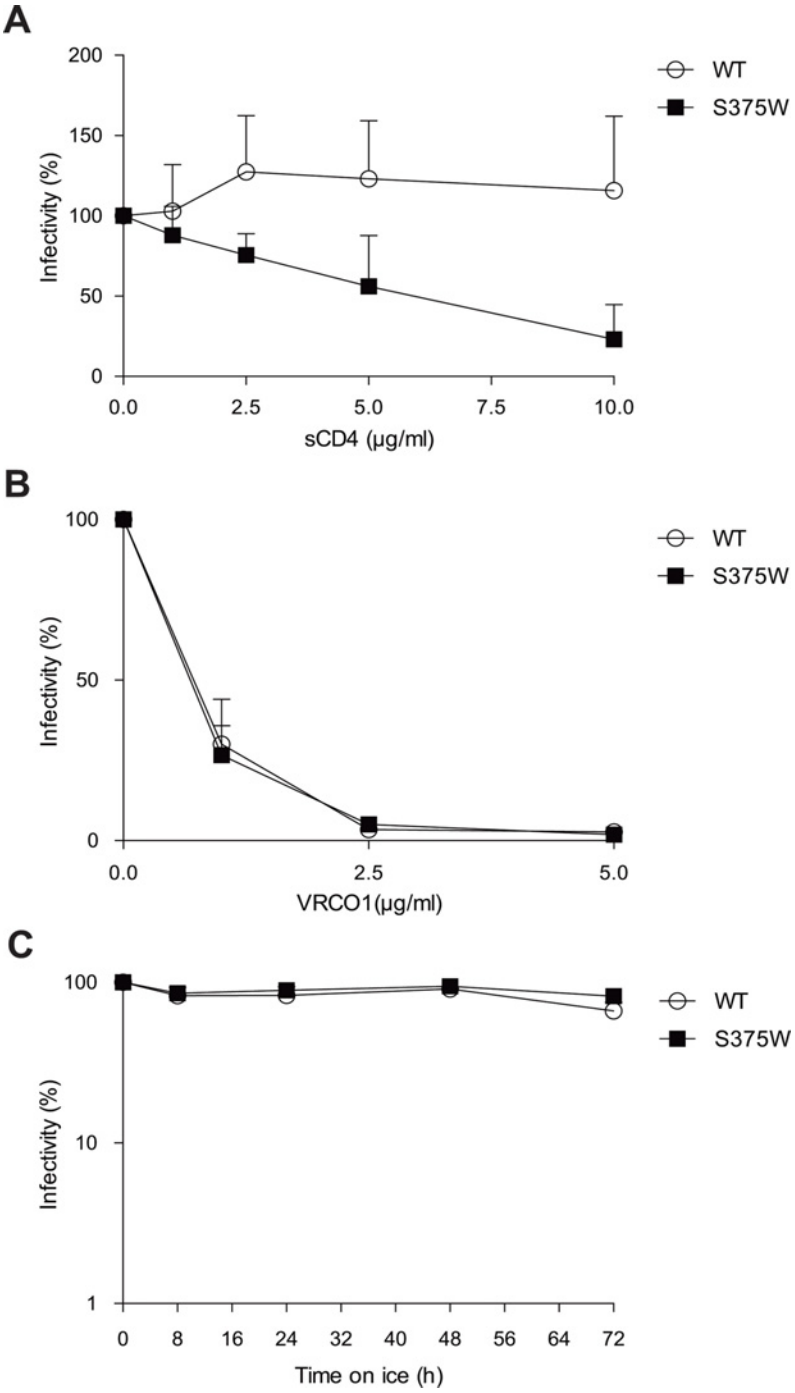

Fig 2. Sensitivity of Env variants to inhibition by sCD4, VRC01 and exposure to cold. Normalized amounts of recombinant luciferase-expressing HIV-1 pseudotyped with the wt and S375W HIV-1_{BG505} Envs were incubated at 37°C with increasing concentrations (0–200 nM) of sCD4 (**A**) or VRC01 (**B**) for 1 hr prior to addition of Cf2Th-CD4/CCR5 cells. In (**C**), recombinant HIV-1 bearing wt or S375W Envs was incubated on ice for different amounts of time [89]. At the indicated time points, aliquots were removed and frozen at -80°C. After completion of the longest incubation, all samples were thawed and infectivity on Cf2Th-CD4/CCR5 cells was measured. Data are representative of results obtained from at least three independent experiments, performed in quadruplicate.

doi:10.1371/journal.pone.0122111.g002

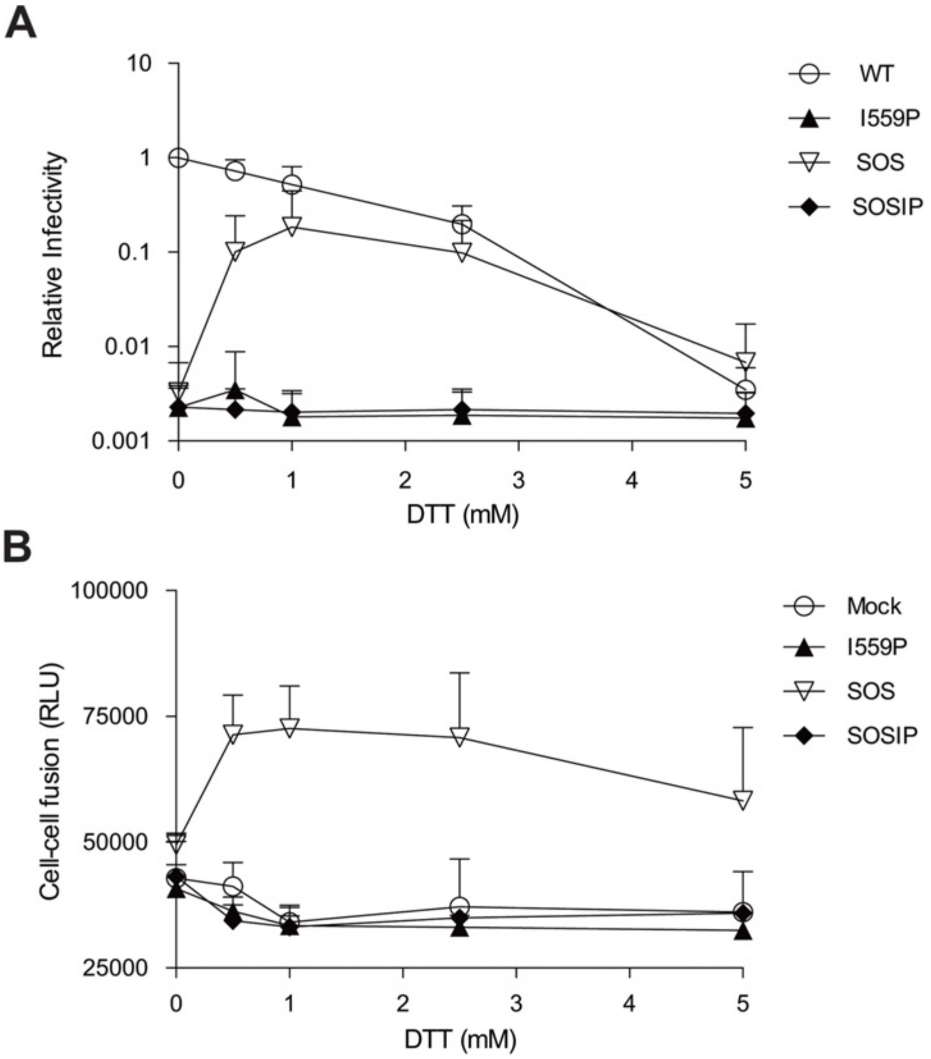

Fig 3. Effect of a reducing agent on the ability of Env variants to mediate infection and cell-cell fusion. (**A**). Normalized amounts of recombinant luciferase-expressing HIV-1 pseudotyped with the wt and mutant HIV-1_{BG505} ΔCT Envs were spin-inoculated onto Cf2Th-CD4/CCR5 cells before adding increasing concentrations of DTT (0–5 mM). (**B**). The ability of the indicated Env variants to mediate cell-cell fusion in the presence of increasing concentrations of DTT was evaluated. The data shown are from a single experiment and are representative of results from at least three independent experiments, performed in quadruplicate.

doi:10.1371/journal.pone.0122111.g003

Ability of Env variants to achieve the CD4-bound state

The recognition of the HIV-1_{BG505} Env variants (wt, S375W, I559P, SOS and SOSIP) by monoclonal antibodies (MAbs) and other ligands was measured by cell-surface ELISA [49,50]. Envs with truncations of the gp41 cytoplasmic tail were used to increase the level of expression on the cell surface. All of the Env variants were expressed on the surface of transfected cells, as determined by binding of the 2G12 MAb, which recognizes a carbohydrate epitope on the gp120 outer domain [60–63] (see Fig 4 and Table 2 legends). The recognition of the Env variants by other Env ligands was normalized to that observed for the 2G12 MAb. The S375W and SOS Envs bound CD4-Ig efficiently, but the binding of CD4-Ig to the I559P mutant was relatively decreased (Fig 5 and Table 2). The spontaneous and sCD4-induced binding of the 17b and A32 MAbs, which recognize distinct CD4-induced (CD4i) gp120 epitopes [59,64,65], to the

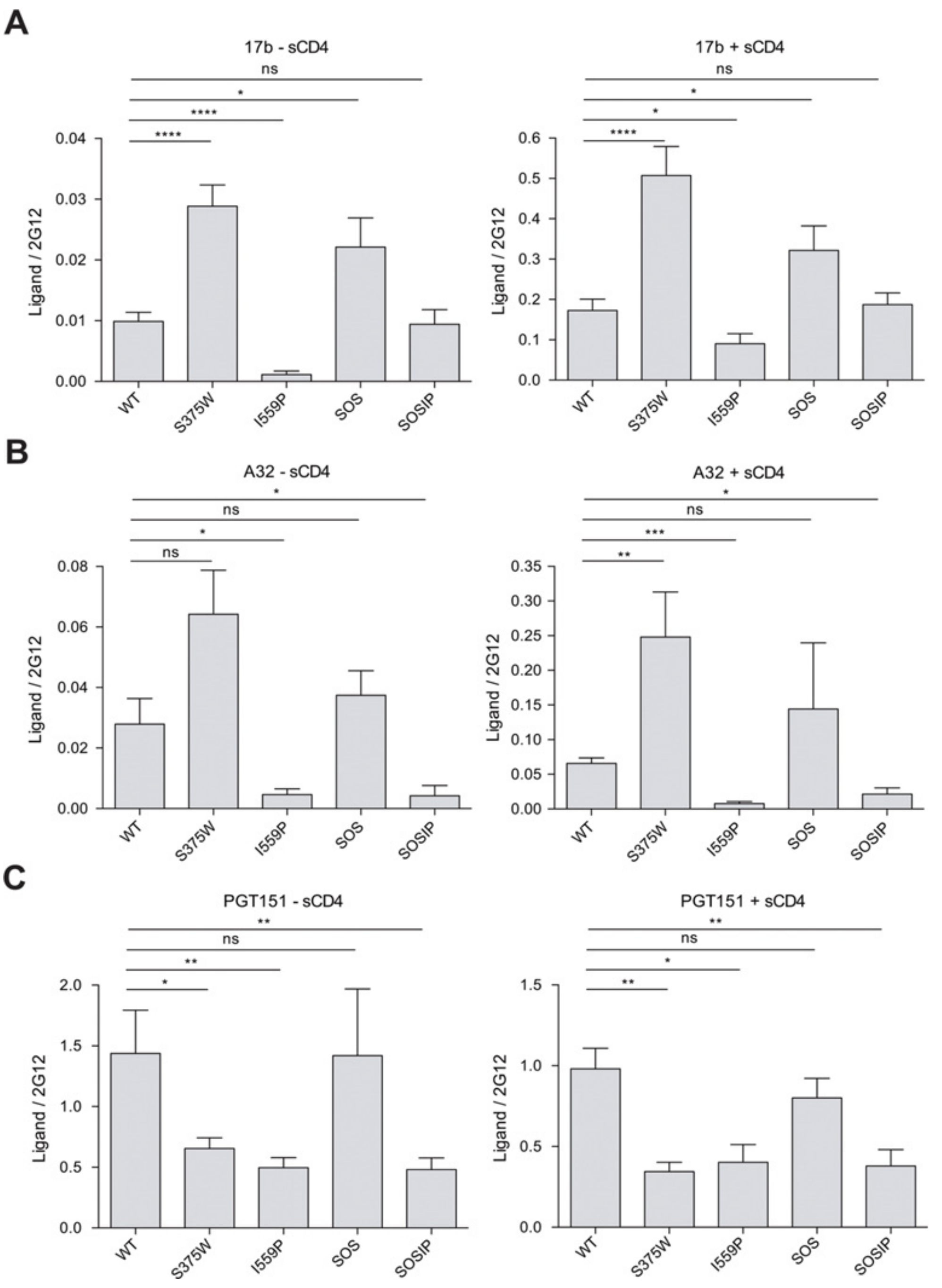

Fig 4. Effect of soluble CD4 on MAb binding to HIV-1_{BG505} Env variants. The binding of 17b (**A**), A32 (**B**) and PGT151 (**C**) MAbs to the indicated HIV-1_{BG505} ΔCT Env variants expressed on the cell surface, in the absence and presence of 80 nM sCD4, is shown. The cell-surface expression of the four Env mutants, as determined by the binding of the 2G12 antibody, was comparable and varied no more than 20% from the level of the wt Env. Each signal was normalized for the relative level of Env expression, using the binding of the 2G12 antibody. Means and SEM derived from at least five independent experiments performed in quadruplicate are shown. Statistical significance is indicated, as described in the Fig 1 legend.

doi:10.1371/journal.pone.0122111.g004

Table 2. Characterization of ligand binding to selected HIV-1$_{BG505}$ variants by cell-based ELISA.[a]

			HIV-1$_{BG505}$ Env variant				
**Env subunit**	**Epitope**	**Ligand**	**wt**	**S375W**	**I559P**	**SOS**	**SOSIP**
gp120	**CD4-BS**	**VRC01**	1.00	0.79	**0.51**	0.82	**0.52**
	**CD4-BS**	**b12**	1.00	**1.72**	**0.19**	1.09	0.73
	**CD4-BS**	**CD4-Ig**	1.00	1.42	**0.31**	1.35	0.74
	**V3**	**19b**	1.00	**1.26**	**0.30**	**1.87**	**0.66**
	**V3**	**GE2 JG8**	1.00	**1.54**	**0.38**	**2.57**	0.96
	**V3 glycans**	**PGT121**	1.00	1.29	**1.73**	1.04	**1.59**
	**V2 glycans**	**PG9 -sCD4**	1.00	0.84	**0.71**	1.01	**0.66**
	**V2 glycans**	**PG9 +sCD4**	0.68	**0.48**	0.61	0.65	0.57
	**CD4i**	**17b -sCD4**	1.00	**3.56**	**<0.01**	**2.68**	1.13
	**CD4i**	**17b +sCD4**	21.80	**61.87**	**12.66**	**35.86**	20.45
	**CD4i**	**A32 -sCD4**	1.00	2.54	**0.03**	1.47	**0.08**
	**CD4i**	**A32 +sCD4**	2.59	**9.81**	**0.19**	5.70	**0.62**
gp120-gp41	**gp120-gp41 interface**	**PGT151 - sCD4**	1.00	**0.45**	**0.34**	0.98	**0.33**
	**gp120-gp41 interface**	**PGT151 + sCD4**	0.68	**0.24**	**0.28**	0.56	**0.26**
	**gp120-gp41 interface**	**35O22 - sCD4**	1.00	**1.47**	1.48	1.00	**1.72**
	**gp120-gp41 interface**	**35O22 + sCD4**	1.02	0.96	**1.84**	1.15	**2.32**
	**gp120-gp41 interface**	**ps[b]**	1.00	0.99	**0.17**	**0.38**	**0.26**
gp41	**MPER**	**4e10**	1.00	1.40	**0.48**	1.24	0.96
	**Cluster I**	**7b2**	1.00	1.08	**0.04**	**0.09**	**0.02**
	**Cluster I**	**F240**	1.00	1.22	**0.03**	**0.25**	**0.07**
	**Cluster II**	**2.2b**	1.00	1.13	**<0.01**	0.55	**0.18**

[a] For the cell-based ELISA experiments, a stop codon was introduced in place of the codon for glycine 711 to truncate the cytoplasmic tail (ΔCT) and enhance cell-surface expression of the indicated HIV-1$_{BG505}$ envelope glycoproteins. Ligand binding was measured by cell-based ELISA, as described in the Materials and Methods. Each signal was normalized for the relative level of Env expression by the binding of the 2G12 antibody. The cell-surface expression of the four Env mutants, as determined by 2G12 binding, was comparable and exhibited no greater than 20% variation from the level of the wt Env. The ligand-binding values relative to those associated with the wt Env in the absence of sCD4 are reported.

[b] The reported values for polyclonal serum (ps) from an HIV-1-infected individual represent the average obtained with serum from one HIV-1-infected individual; however, similar results were obtained with sera from more than six randomly selected HIV-1-infected individuals.

Values presented in this table represent the mean of at least four independent experiments done in quadruplicate, with experimental variation typically not more than 20% of the value reported. In bold are values that are statistically different ($p \leq 0.05$) from those of the wt Env, as measured by a paired *t* test.

doi:10.1371/journal.pone.0122111.t002

S375W mutant was increased, relative to that observed for wt Env (Fig 4 and Table 2). Consistent with the results obtained with Envs from other HIV-1 strains [49,54,56–58], filling the Phe43 cavity with a tryptophan residue apparently allows more efficient sampling of the CD4-bound conformation by the HIV-1$_{BG505}$ gp120 core.

The spontaneous recognition of the I559P mutant by the 17b and A32 MAbs was dramatically reduced, compared with that of the wt Env (Fig 4 and Table 2). The binding of sCD4 partially restored 17b binding to the I559P Env, but A32 binding to the I559P mutant was significantly decreased compared to wt Env, even in the presence of sCD4. These results suggest that the sampling of the CD4-bound conformation by the gp120 subunit is reduced by the I559P change in gp41.

Spontaneous and sCD4-induced binding of the 17b MAb was greater for the SOS mutant than for the wt Env (Fig 4 and Table 2). Moreover, the SOSIP mutant bound the 17b MAb comparably to wt Env, in both the absence and presence of sCD4. Apparently, the SOS change results in a greater propensity to form and expose the 17b epitope on gp120, which can compensate for

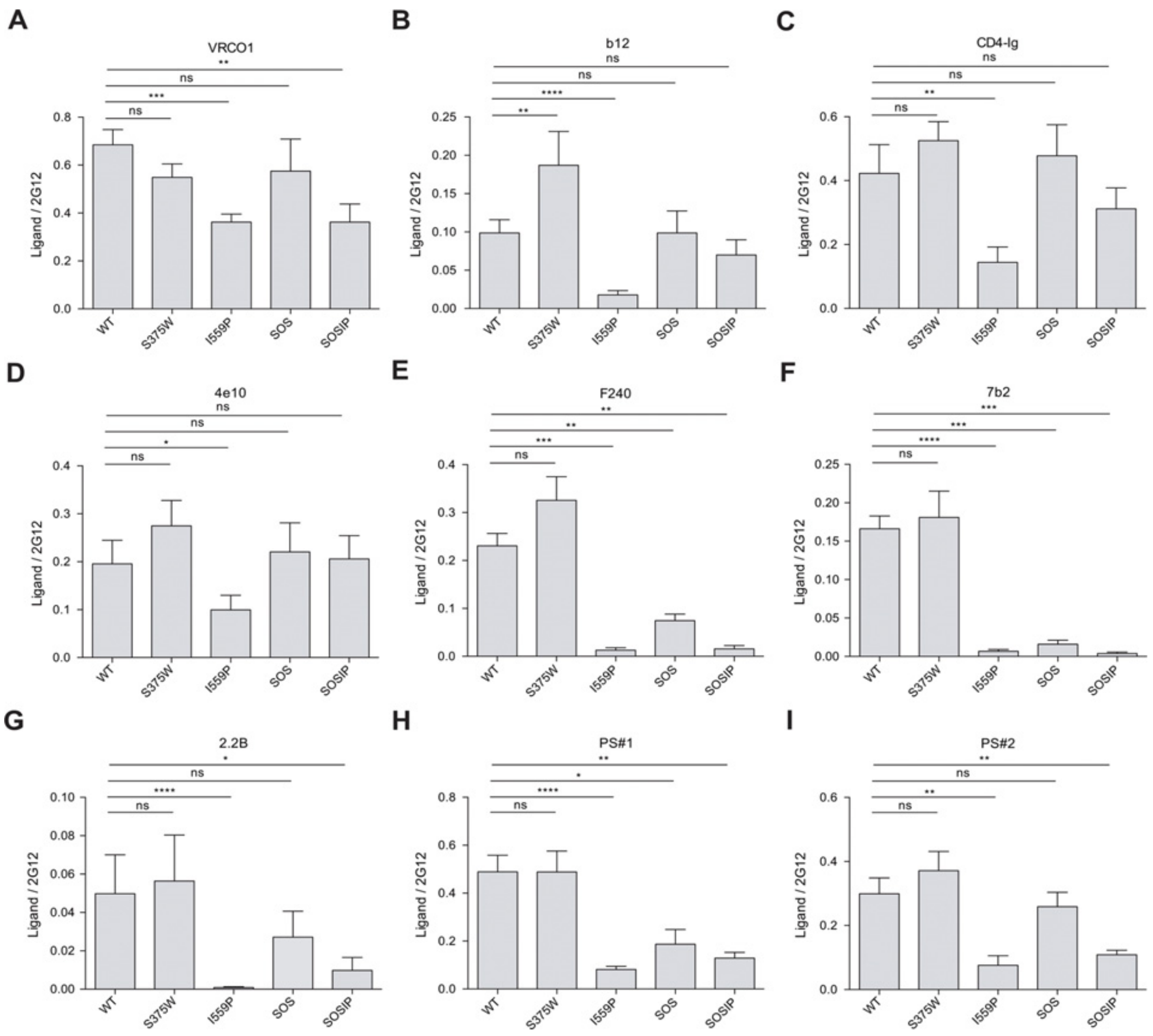

Fig 5. Binding of MAbs and sera from HIV-1-infected individuals to Env variants. The binding of VRC01 (**A**), b12 (**B**), CD4-Ig (**C**), 4e10 (**D**), F240 (**E**), 7b2 (**F**), 2.2B (**G**) or sera from HIV-1-infected individuals (patient sera, PS#1) (**H**) and PS#2 (**I**) to HIV-1_{BG505} ΔCT Env variants expressed on the cell surface is shown. Means and SEM derived from at least five independent experiments performed in quadruplicate are shown. Statistical significance is indicated, as described in the Fig 1 legend.

doi:10.1371/journal.pone.0122111.g005

the negative effects of the I559P alteration on 17b binding. By contrast, similar to the results with the I559P mutant, A32 binding to SOSIP Env in the absence or presence of sCD4 was significantly lower than that of the wt Env. Thus, some but not all of the inhibitory effects of the I559P gp41 change on gp120 transitions to the CD4-bound state can be compensated by the SOS modification.

Evaluation of the conformation of Env variants with an antibody panel

Antibody recognition of the cell-surface wt and mutant Envs was evaluated by the cell-based ELISA (Fig 5 and Table 2). The recognition of the I559P Env by the CD4-binding site (CD4BS) MAbs, VRC01 and b12, was decreased relative to that of the wt Env. Decreased binding of b12 to the I559P mutant Env from HIV-1_{JR-FL} has been previously noted [66]. Although b12 binding

to the SOSIP Env was better than that of the I559P Env, VRC01 binding to the SOSIP mutant was significantly decreased compared with wt Env. Apparently, the I559P and SOS changes can alter Env quaternary structure so that MAb binding to the CD4-binding site of gp120 is affected. Consistent with this explanation, recognition of the I559P and SOSIP Envs by the PG9 MAb, which recognizes gp120 V2 region glycans in a manner that is sensitive to quaternary trimer conformation [67], was slightly lower than that of the wt Env (Table 2 and Fig 6).

The PGT121 neutralizing MAb, which recognizes a carbohydrate-dependent epitope in the gp120 V3 region [68,69], bound the I559P and SOSIP mutants better than the wt Env (Table 2). By contrast, the 19b and GE2 JG8 anti-V3 MAbs bound the I559P variant less efficiently than the wt Env (Fig 7 and Table 2); similar results were also obtained with the 447 D52 and 2191 anti-V3 MAbs (data not shown). Decreased 19b but not GE2 JG8 binding was also observed for the SOSIP mutant. Both anti-V3 MAbs recognized the SOS mutant more efficiently than the wt Env. These results further suggest that the I559P change in gp41 alters the gp120 conformation in the Env trimer.

The binding of two neutralizing MAbs, PGT151 and 35O22, which recognize gp120-gp41 hybrid epitopes [70–72], to the cell-surface Envs was evaluated in the absence and presence of sCD4 (Table 2). The binding of PGT151 to the S375W, I559P and SOSIP Env mutants was reduced, relative to that of the wt and SOS Envs. Incubation with sCD4 reduced PGT151 binding to all of the Env variants. The 35O22 MAb bound better to the S375W and SOSIP Envs than to the wt Env in the absence of sCD4. In the presence of sCD4, the I559P and SOSIP Envs exhibited a higher-than-wt level of 35O22 MAb binding. These results suggest that the S375W, I559P and SOSIP changes lead to alterations in the relationship between the gp120 and gp41 subunits of the Env trimer.

Additional MAbs directed against gp41 epitopes were tested for recognition of the wt and mutant Envs. MAbs against gp41 Cluster I and Cluster II epitopes exhibited much lower binding to the I559P mutant compared with the wt Env. The relative binding of these MAbs to the SOSIP mutant was also low, indicating that the SOS alteration does not compensate for the I559P change in this instance. In fact, the recognition of the SOS mutant by the Cluster I MAbs was lower than that of the A501C and T605C single-cysteine substitution mutants (data not shown), suggesting that both cysteines may need to form a disulfide bond to cause the observed reduction in recognition of the SOS mutant. A neutralizing MAb directed against the gp41 membrane-proximal external region (MPER), 4E10, exhibited a reduction in recognition of the I559P mutant relative to that of the wt Env. Thus, the I559P change significantly alters the conformation of gp41 in a manner that is minimally compensated by the SOS modification.

We also examined the recognition of the wt and mutant Envs on the cell surface by polyclonal sera from HIV-1-infected individuals. Remarkably, the I559P and SOSIP Envs were recognized less efficiently than the wt and S375W Envs by sera from several HIV-1-infected individuals (Table 2 and Fig 5). Modest decreases in the binding of these sera to the SOS mutant, relative to that of wt Env, were also noted. Thus, the Env complexes with the I559P changes exhibit significant changes in the surface exposure of epitopes recognized by antibodies generated in a subset of HIV-1-infected subjects.

Several previous studies have suggested that the long cytoplasmic tail of the HIV-1 Env can influence the structure of the Env ectodomain [42,73–75]. In the cell-based ELISA experiments described above, the Env cytoplasmic tail was truncated to enhance Env expression at the cell surface. To test whether the dramatic effects of the I559P change on Env conformation (Fig 8 and Table 2) were dependent on the truncation of Env, we performed additional cell-based ELISA experiments using the full-length HIV-1_{BG505} gp160 envelope glycoproteins. As shown in Fig 9, the introduction of the I559P change into the full-length gp160 Env closely recapitulated the profound conformational effects described above for Envs lacking the cytoplasmic

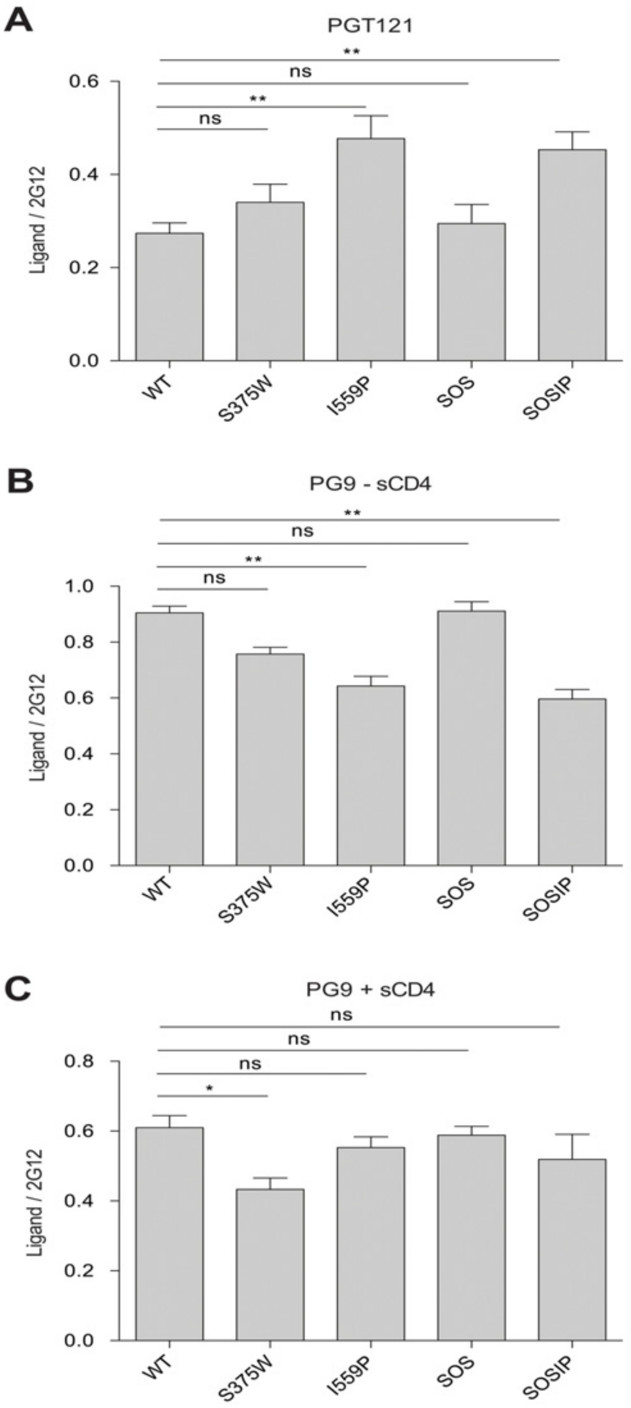

Fig 6. MAb binding to HIV-1$_{BG505}$ Env variants on the cell surface. The binding of PGT121 (**A**) and PG9 (**B** and **C**) to HIV-1$_{BG505}$ Env ΔCT variants expressed on the cell surface was measured by cell-based ELISA. The data in (**C**) were generated in the presence of 80 nM sCD4. The means and SEM derived from at least three independent experiments performed in quadruplicate are shown. Statistical significance was assessed using a paired t-test, and is indicated as described in the legend to Fig 1 of the main text.

doi:10.1371/journal.pone.0122111.g006

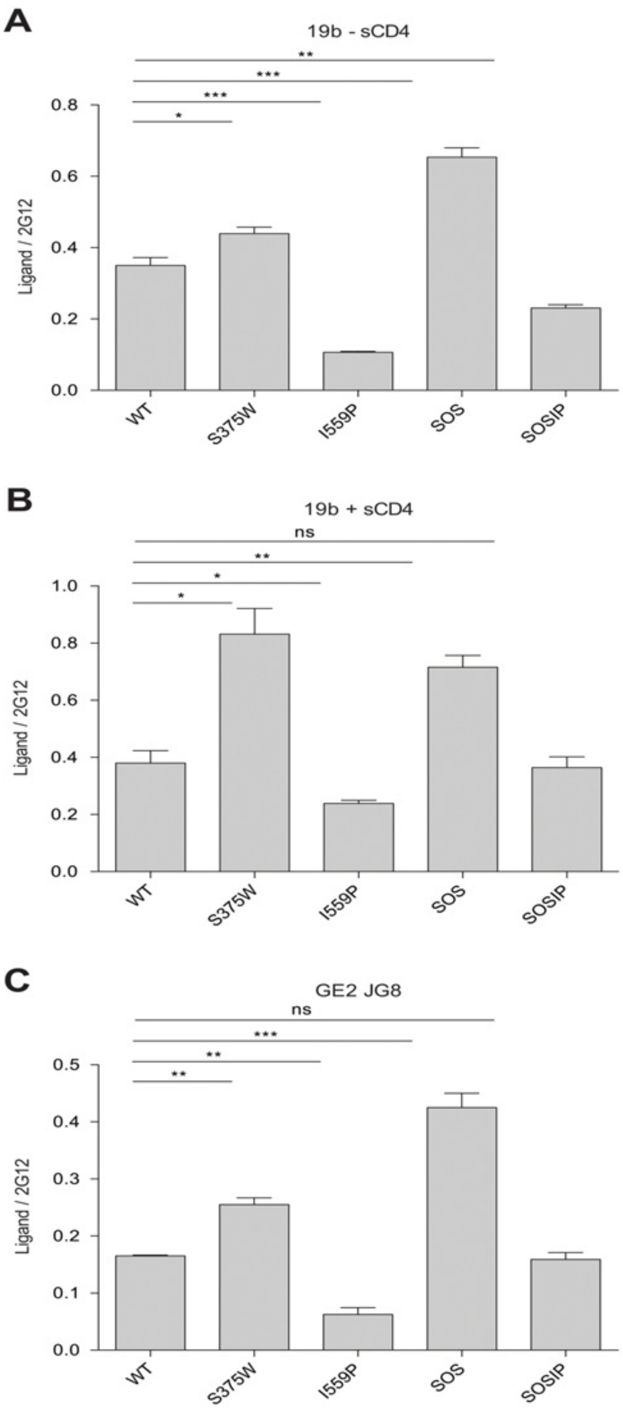

Fig 7. Anti-V3 MAb binding to HIV-1$_{BG505}$ Env variants on the cell surface. The binding of the indicated MAbs to HIV-1$_{BG505}$ Env ΔCT variants expressed on the cell surface was measured by cell-based ELISA. The means and SEM derived from at least three independent experiments performed in quadruplicate are shown. Statistical significance was assessed using a paired t-test, and is indicated as described in the legend to Fig 1 of the main text.

doi:10.1371/journal.pone.0122111.g007

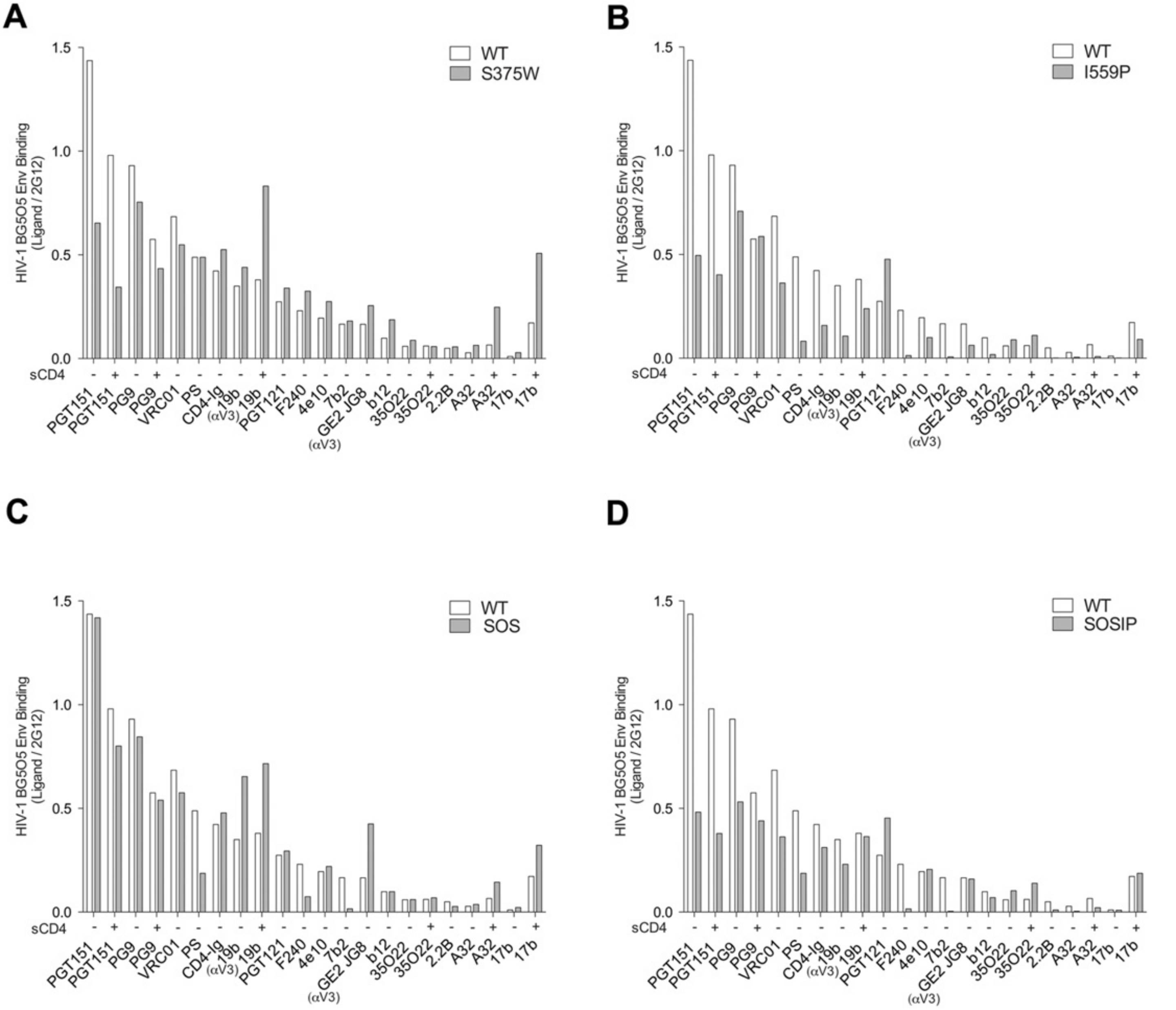

Fig 8. Ligand binding to HIV-1$_{BG505}$ Env variants. The binding of each of the indicated ligands to the HIV-1$_{BG505}$ ΔCT Env variants expressed on the cell surface was measured by the cell-based ELISA, and is plotted relative to the binding of the 2G12 antibody. In some cases, ligand binding was measured in the presence of 80 nM sCD4. The ligands are arranged according to the relative binding to wt Env in the absence of sCD4.

doi:10.1371/journal.pone.0122111.g008

tail. Indeed, the I559P change resulted in decreased full-length Env recognition by the CD4BS MAbs, VRC01 and b12. Compared with the wt full-length Env, the I559P mutant was recognized less efficiently by the neutralizing MAb, PGT151, by the F240 anti-gp41 MAb, and by sera from HIV-1-infected individuals. Thus, the major conformational changes induced by the I559P change in the membrane-anchored Env are independent of the presence of the gp41 cytoplasmic tail.

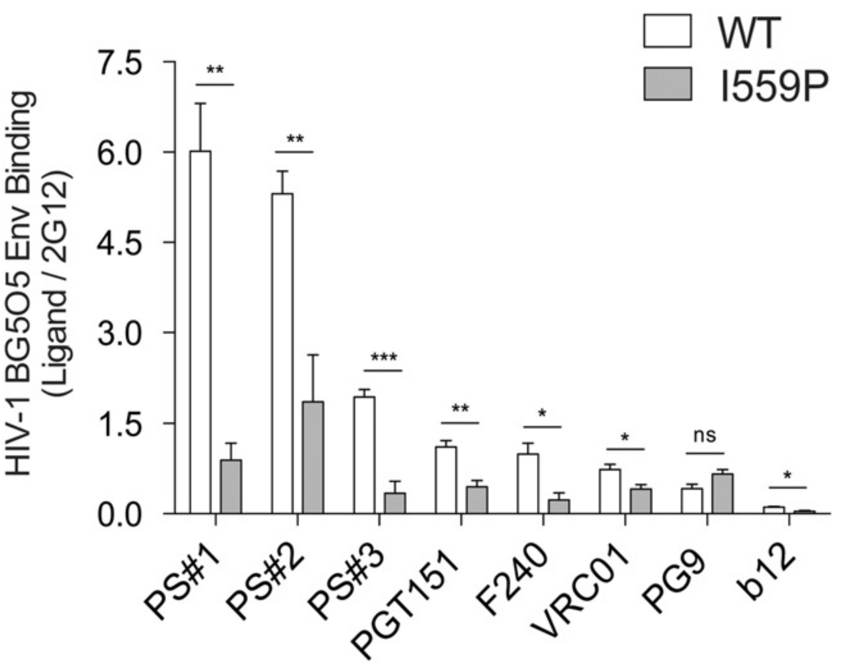

Fig 9. Ligand binding to HIV-1$_{BG505}$ full-length wild-type and I559P Env variants. The binding of wild-type (wt) and I559P HIV-1$_{BG505}$ Env full-length (gp160) variants expressed on the cell surface was measured by cell-based ELISA, and is plotted relative to the binding of the 2G12 antibody. The ligands are arranged according to the relative binding to wt Env. The means and SEM derived from four independent experiments performed in quadruplicate are shown. Statistical significance was assessed using a paired t-test, and the degree of significance is indicated: ***—$p < 0.001$, **—$p < 0.01$, *—$p < 0.05$ and ns—not significant.

doi:10.1371/journal.pone.0122111.g009

Discussion

The unliganded HIV-1 Env trimer on the viral membrane must retain a high level of potential energy to function as a membrane-fusing machine, and thereby exists in a metastable state [1,76,77]. Whereas binding to the receptors on the target cell membrane triggers Env movement into lower-energy, on-pathway conformations, other influences (amino acid changes, ligand binding, exposure to cold) can lead to more stable Env conformations that are irreversibly inactivated [49,56,76,77]. In creating more stable HIV-1 Env trimers for structural studies, information about the phenotypic consequences of the introduced Env amino acid changes can help to assess whether and how the observed structures fit into the virus entry pathway. Introduction of the helix-breaking proline at residue 559 in gp41 was intended to disrupt the formation of the HR1 coiled coil in the prehairpin intermediate [30]. The I559P change was found to stabilize the formation of trimers in the otherwise heterogeneous soluble gp140 Envs [30,34,35]. All HIV-1 Env trimer preparations currently analyzed by x-ray crystallography contain the I559P change and the SOS alteration that covalently links gp120 and gp41, as well as deletions of part of the gp41 ectodomain, transmembrane region and cytoplasmic tail [37,38]. We investigated the impact of the I559P change, with and without the SOS changes, on the conformation and function of the membrane-anchored Env from HIV-1$_{BG505}$, the same strain of origin as that of the soluble gp140 SOSIP.664 trimer used in most structural studies [36–38,70–72,78–80].

The phenotypes observed in this study suggest that the I559P mutant differs from wt Env in the conformations of both the gp120 and gp41 subunits. The association of gp120 with the I559P Env trimer was tighter than that observed for wt Env, suggesting that the I559P change

alters the relationship among the gp120 and gp41 subunits. Consistent with this, the PGT151 neutralizing MAb, which recognizes a hybrid gp120-gp41 epitope [71,72], bound the I559P Env less efficiently than wt Env. The 35O22 MAb, which recognizes a different gp120-gp41 hybrid epitope [70], bound more efficiently to the I559P mutant than to wt Env in the presence of sCD4. The binding of several ligands (CD4-Ig, VRC01, b12) that recognize a gp120 region near the CD4-binding site was decreased to the I559P mutant, relative to the values seen for wt Env. As these ligands must bypass the adjacent protomer to engage their epitopes [42,81], their binding may be sensitive to differences in quaternary conformation (Fig 10). Recognition of the I559P and SOSIP Envs by the PG9 MAb, whose epitope can be influenced by quaternary

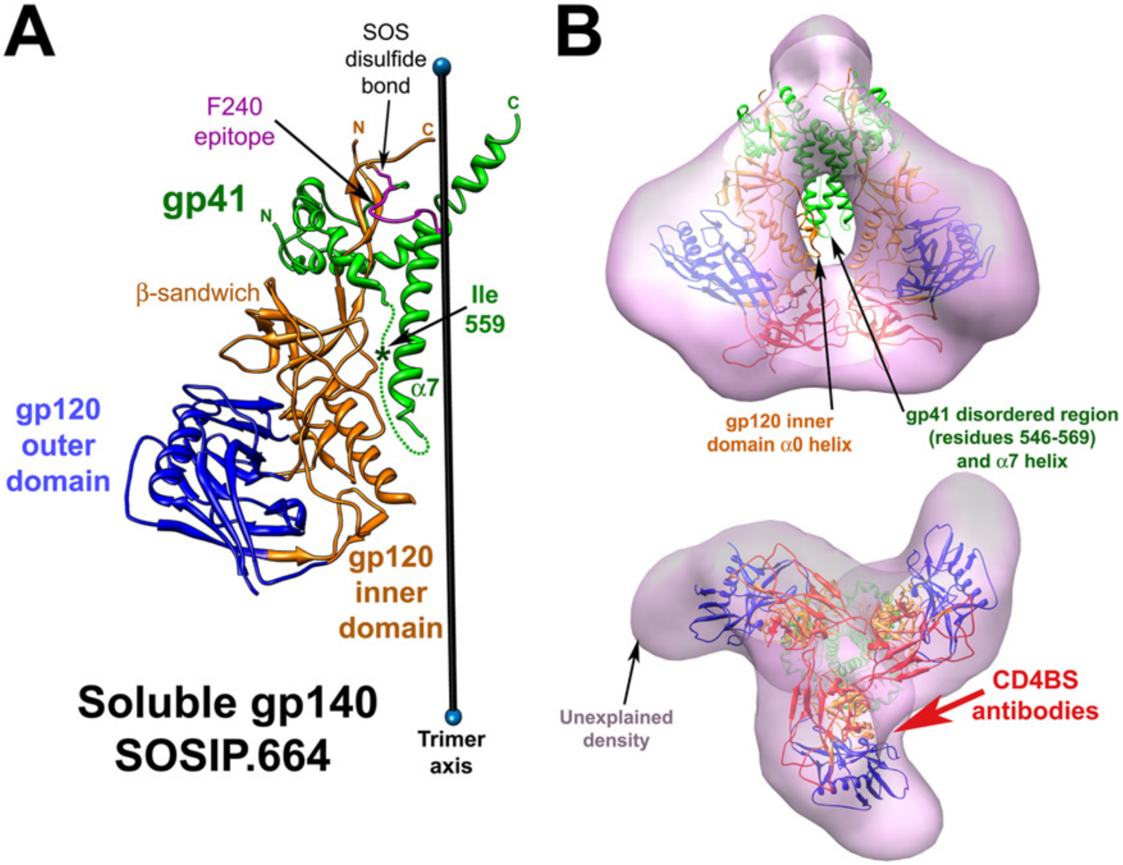

Fig 10. Insights into the I559P phenotypes from the structure of the soluble gp140 SOSIP.664 Env trimer. (**A**). The ribbon structure (PDB 4TVP) of one protomer of the HIV-1$_{BG505}$ soluble gp140 SOSIP.664 Env trimer [38] is shown, oriented with gp41 at the top of the figure. The gp41 subunit is colored green, the gp120 outer domain is colored blue, and the gp120 inner domain and N- and C-termini are colored orange. The approximate location of isoleucine 559 within the disordered gp41 segment (residues 548–568) (dotted line) is indicated by an asterisk. The gp41 α7 helix forms a coiled coil at the trimer axis. The SOS disulfide bond linking gp120 residue 501 and gp41 residue 605 is labeled. The F240 Cluster I gp41 epitope is highlighted in magenta, and is buried near the trimer axis in the soluble gp140 SOSIP.664 structure. The gp120 core is in the CD4-bound conformation, except for the V1/V2 stem, which is not shown. The β-sandwich of the gp120 inner domain, which is involved in the non-covalent association of gp120 and gp41 [90], loosely interacts with the gp41 fusion peptide in this structure. (**B**). The soluble SOSIP.664 Env trimer structure [38] was fitted as a rigid body into the tomogram of the HIV-1 virion Env trimer (grey) (EMD 5019) [40], using UCSF Chimera software [91–93]. The soluble gp140 SOSIP.664 ribbon structure is colored as in **A** and, in addition, the structure of the gp120 V1/V2 and V3 variable regions is shown in red. In the upper panel, the Env spike is oriented such that the viral membrane is at the top of the image. The gp41 disordered region (residues 548–568), including isoleucine 559, and the α7 helix of the soluble gp140 SOSIP.664 structure are out of the virion Env density, as is the α0 helix of the gp120 inner domain. The lower panel shows a view from the perspective of the target cell. Note the density in the gp120 arms of the virion Env tomograms that is not explained by the soluble gp140 SOSIP.664 structure. The approximate angle of approach used by the CD4-binding site (CD4BS) antibodies to engage the HIV-1 Env trimer is indicated by the red arrow. Because these antibodies must access their epitopes by bypassing the adjacent gp120 protomer, their binding is potentially sensitive to changes in the orientation of the gp120 subunits in the quaternary Env trimer structure [42,81].

doi:10.1371/journal.pone.0122111.g010

Env structure [67], was mildly decreased relative to that of the wt Env. Consistent with an alteration in the gp120-gp41 relationship in the I559P Env, the I559P change in another HIV-1 Env has been reported to result in a loss of sCD4-induced gp120 shedding [66].

The original rationale for the introduction of the I559P change was to destabilize the CD4-bound pre-hairpin intermediate, hopefully predisposing Env to remain in the unliganded state [30]. Indeed, the spontaneous sampling of the CD4-bound conformation by the gp120 subunit appeared to be decreased for the I559P mutant, relative to that of the wt HIV-1_{BG505} Env. In the absence of sCD4, binding of the 17b and A32 MAbs, which prefer the CD4-bound state of gp120 [59,64,65], was reduced to background levels by the I559P change. The I559P change also reduced the binding of several MAbs directed against the gp120 V3 region, another Env element that is exposed better in the CD4-bound state. That an alternation of gp41 influences the conformational states of gp120 suggests an interdependency of the subunits within the Env trimer. For some (17b, GE2 JG8, b12, CD4-Ig) but not all of the gp120-directed ligands, the observed decrease in binding to the I559P Env was compensated by the SOS alteration. It is noteworthy that the SOSIP mutant bound the 17b antibody as well as the wt Env, even in the absence of sCD4. This observation argues against a model in which the I559P and SOS changes lock Env in an unliganded state. Thus, while the I559P mutant resists movement into the CD4-bound state, the I559 change does not guarantee that Env retains an unliganded conformation.

Dramatic reductions in the binding of gp41-directed MAbs were observed for the I559P Env, compared with that of wt Env. The mildly decreased processing of the I559P mutant relative to that of wt Env is expected to increase the exposure of the Cluster I and Cluster II gp41 epitopes [82], so the reduced binding of MAbs to these epitopes is all the more striking. With the exception of the mild decrease in recognition by the 4E10 MAb, the relative decrease in anti-gp41 MAb binding to the I559P Env was not compensated by the SOS change.

Compared with the wt HIV-1_{BG505} Env, the I559P Env mutant was recognized inefficiently by the polyclonal sera from several HIV-1-infected individuals. These sera are known to contain antibodies against multiple epitopes on both gp120 and gp41. These observations support the conclusion that the I559P change causes significant conformational changes in the gp120 and gp41 subunits within the Env trimer. Importantly, the I559P change induced profound conformational changes within both cytoplasmic-tail truncated (Fig 8) and full-length (Fig 9) Envs. Thus, the I559P modification alters Env structure in a manner that is independent of the presence of the cytoplasmic tail, some changes in which have been previously shown to affect the structural and functional properties of the Env ectodomain [42,73–75].

The relative efficiency of binding of each Env ligand to the wt and mutant HIV-1_{BG505} Envs is shown in Fig 8. With a few exceptions (PGT121 and 35O22), the I559P change was associated with decreased recognition by the Env ligands. Importantly, decreased binding of the potent neutralizing antibodies VRC01 and PGT151 is incompatible with a model in which I559P stabilizes the unliganded state of Env. As the broad and potent neutralizing antibodies generally bind more efficiently than non-neutralizing antibodies to the wt Env [76], the epitopes for the potent neutralizing antibodies tended to be preserved on the I559P-containing Envs. In agreement with our data on the membrane SOSIP Env, SOSIP-stabilized soluble gp140 Env trimers retain multiple epitopes for potent, broadly neutralizing antibodies, but are recognized less efficiently by weakly neutralizing or non-neutralizing antibodies [34]. This pattern of epitope exposure could be a desirable quality in an immunogen aimed at eliciting neutralizing antibodies. However, because many potent and broad neutralizing antibodies, particularly those recognizing glycans, tolerate variation in their Env epitopes [61,83–85], they may be less sensitive indicators of conformational disruption than weakly neutralizing antibodies. Therefore, retention

of the epitopes for broadly neutralizing antibodies cannot be equated with maintenance of a native functional Env state.

How might the I559P change alter the conformation of the membrane-anchored HIV-1_{BG505} Env? In the crystal structure of the HIV-1_{BG505} soluble gp140 SOSIP.664 trimer, the gp41 region surrounding isoleucine 559 (residues 548–568) is disordered [38] (Fig 10A). As this gp41 region is highly conserved in HIV-1 and is critical for mediating the non-covalent association with gp120 [86], this disorder is unexpected and unlikely to exist in the native unliganded Env (see below). Although Env modifications besides I559P may have contributed to the structure of the soluble gp140 SOSIP.664 glycoprotein observed in the crystal, the isoleucine 559 change is the only alteration within the disordered gp41 segment and thus is a reasonable candidate for the source of this disorder. It is possible that the I559P change results in the disruption of the native gp120-gp41 interface in the context of the membrane-anchored Env and could account for the diverse effects of the I559P change on gp120 and gp41 conformation observed in this and other studies [66].

The I559P change completely eliminated the ability of the primary HIV-1_{BG505} Env to mediate syncytium formation and virus entry, as has been previously observed in the context of a laboratory-adapted HIV-1 Env [87]. Moreover, although the infectivity of the SOS mutant could be rescued by exposure to the reducing agent DTT, the SOSIP variant containing the I559P change was non-functional regardless of the redox environment. Thus, there currently is no evidence that an HIV-1 Env with an I559P change can mediate membrane fusion or support virus entry. At a minimum, the helix-disrupting I559P substitution should impede the formation of the gp41 HR coiled coil [30]. As the antigenic conformation of the I559P Env is not consistent with that expected for the unliganded state of Env (see above), the I559P change may exert other effects that diminish Env function. The relationship between structures of Envs with the I559P alteration and conformations of the functional HIV-1 Env spike remains to be determined.

How might the I559P change stabilize soluble gp140 SOS trimers? With the goal of stabilizing an unliganded state of the soluble gp140 Env trimers, the I559P alteration was intended to disrupt the gp41 HR1 helix and thereby destabilize the downstream pre-hairpin intermediate [30]. The success of this strategy in creating more stable soluble trimers in the unliganded conformation relies on two untested assumptions: 1) that the trimeric associations in the unliganded state are tighter than those in the pre-hairpin intermediate, and 2) that the I559P change does not disrupt the unliganded Env conformation more than that of the pre-hairpin intermediate. The extensive scope of the conformational consequences of the I559P change documented in our study calls into question the latter assumption. This raises the possibility that I559P stabilizes the soluble gp140 SOS trimers by a means other than locking Env in the unliganded state. This possibility is supported by the examination of the crystal structure of the soluble gp140 SOSIP.664 trimer [38] fitted into the tomograms of the native unliganded Env on virions [40] (Fig 10B, upper panel). Two gp41 regions of the soluble gp140 SOSIP.664 trimer are outside of the tomographic density and are therefore incompatible with a native unliganded state of Env. The two gp41 regions are: 1) the long disordered region (residues 548–568) that includes isoleucine 559; and 2) the α7 helix, which forms a trimer-stabilizing coiled coil at the trimer axis (Fig 10A). These two gp41 elements are highly conserved among HIV-1 isolates, so strain variation is unlikely to account for this incompatibility. Instead, we hypothesize that the I559P change disrupts the conformation of the gp41 ectodomain in the soluble gp140 SOSIP.664 trimer and indirectly leads to the formation of the trimer-stabilizing α7 helical coiled coil at the trimer axis. This model resolves the dilemma of explaining stabilization of the soluble gp140 SOSIP.664 trimer by a change in a disordered gp41 region. This model is compatible with two other observations: 1) an α-helical bundle distant from the

trimer axis exists in the gp41 ectodomain of an unliganded immature Env trimer [42]; and 2) the substitution of helix-breaking residues (proline and glycine) for isoleucine 559 stabilizes the soluble gp140 trimers better than other amino acid substitutions [30]. Thus, during the folding of the soluble gp140 SOSIP.664 Env, the I559P change might disrupt the native gp41 α-helical bundle, causing the α7 helix and the newly disordered region around isoleucine 559 to collapse towards the trimer axis. This model explains the observed differences between the crystal structure of the soluble gp140 SOSIP.664 trimer [38] and the tomograms of the native unliganded Env spike [40] (Fig 10B, upper panel). The density in the distal arms of the virion Env trimer [40] that is not explained by the soluble gp140 SOSIP.664 structure [37,38] (Fig 10B, lower panel) may reflect the shift of the gp120 subunits towards the trimer axis expected as a consequence of the I559P-induced changes. Indeed, part of the gp120 inner domain in the soluble gp140 SOSIP.664 structure [37,38] abuts the trimer axis and falls outside the density map of the native virion Env spike [40] (Fig 10B, upper panel).

In the crystal structure of the HIV-1_{BG505} soluble gp140 SOSIP.664 trimer, the gp120 core (inner domain, outer domain and bridging sheet) is in the CD4-bound conformation [37,38]. The CD4-bound conformation is a default state assumed by the HIV-1 gp120 core when the constraints operative in the unliganded state are removed [88]; in the soluble gp140 SOSIP.664 structure, the CD4-bound state of the gp120 core may have resulted from a disruption by I559P of the native gp41-mediated constraints on the unliganded gp120 conformation. To investigate the conformation of the gp120 core in the functional HIV-1_{BG505} Env trimer, we introduced a tryptophan residue into the Phe43 cavity, which predisposes the gp120 core to assume the CD4-bound conformation [54,55]. Compared with the wt HIV-1_{BG505} Env, the S375W mutant exhibited greater sensitivity to neutralization by sCD4, but not by the VRC01 CD4BS MAb. The S375W Env bound antibodies like 17b and A32, which prefer the CD4-bound conformation [59,64,65], more efficiently than the wt HIV-1_{BG505} Env, particularly in the presence of sCD4. These results suggest that, like other functional HIV-1 Envs examined to date [40,49,54,56–58,76,77,88], the unliganded HIV-1_{BG505} Env exists in a metastable state with a gp120 core distinct from that found in the CD4-bound state. Thus, the appearance of a CD4-bound gp120 core conformation in the HIV-1_{BG505} soluble gp140 SOSIP.664 structure [37,38] suggests that this structure is unlikely to represent the native unliganded state of Env. Might the soluble gp140 SOSIP.664 structure [37,38] represent another HIV-1 Env conformation on the entry pathway? The soluble gp140 SOSIP.664 structure is clearly incompatible with tomograms of the virion Env spike in its "open," CD4-bound conformation [40]. A proper assessment of the relationship of the soluble gp140 SOSIP.664 structure to the HIV-1 entry pathway will require additional data. Future efforts to define the structure of the HIV-1 Env trimer in its unliganded and receptor-bound states should recognize and address its natural membrane environment and metastability.

Acknowledgments

We thank Ms. Yvette McLaughlin and Ms. Elizabeth Carpelan for manuscript preparation. We thank Dr. Alon Herschhorn for sharing the pcDNA3.1-BG505 plasmid. We thank Dr. James Robinson for kindly providing the 19b mAb, and Dr. Gunilla Karlsson Hedestam and Ganesh Phad for providing the GE2 JG8 MAb. Sera from HIV-1-infected individuals were obtained from the FRQS-SIDA network and we thank Mario Legault for cohort coordination.

Author Contributions

Conceived and designed the experiments: AF JS. Performed the experiments: NA OD. Analyzed the data: NA OD JS AF. Wrote the paper: JS AF.

References

1. Wyatt R, Sodroski J (1998) The HIV-1 envelope glycoproteins: fusogens, antigens, and immunogens. Science 280: 1884–1888. PMID: 9632381
2. Allan JS, Coligan JE, Barin F, McLane MF, Sodroski JG, et al. (1985) Major glycoprotein antigens that induce antibodies in AIDS patients are encoded by HTLV-III. Science 228: 1091–1094. PMID: 2986290
3. Robey WG, Safai B, Oroszlan S, Arthur LO, Gonda MA, et al. (1985) Characterization of envelope and core structural gene products of HTLV-III with sera from AIDS patients. Science 228: 593–595. PMID: 2984774
4. Chan DC, Fass D, Berger JM, Kim PS (1997) Core structure of gp41 from the HIV envelope glycoprotein. Cell 89: 263–273. PMID: 9108481
5. Weissenhorn W, Dessen A, Harrison SC, Skehel JJ, Wiley DC (1997) Atomic structure of the ectodomain from HIV-1 gp41. Nature 387: 426–430. PMID: 9163431
6. Lu M, Blacklow SC, Kim PS (1995) A trimeric structural domain of the HIV-1 transmembrane glycoprotein. Nat Struct Biol 2: 1075–1082. PMID: 8846219
7. Tan K, Liu J, Wang J, Shen S, Lu M (1997) Atomic structure of a thermostable subdomain of HIV-1 gp41. Proc Natl Acad Sci U S A 94: 12303–12308. PMID: 9356444
8. Klatzmann D, Champagne E, Chamaret S, Gruest J, Guetard D, et al. (1984) T-lymphocyte T4 molecule behaves as the receptor for human retrovirus LAV. Nature 312: 767–768. PMID: 6083454
9. Dalgleish AG, Beverley PC, Clapham PR, Crawford DH, Greaves MF, et al. (1984) The CD4 (T4) antigen is an essential component of the receptor for the AIDS retrovirus. Nature 312: 763–767. PMID: 6096719
10. Wu L, Gerard NP, Wyatt R, Choe H, Parolin C, et al. (1996) CD4-induced interaction of primary HIV-1 gp120 glycoproteins with the chemokine receptor CCR-5. Nature 384: 179–183. PMID: 8906795
11. Trkola A, Dragic T, Arthos J, Binley JM, Olson WC, et al. (1996) CD4-dependent, antibody-sensitive interactions between HIV-1 and its co-receptor CCR-5. Nature 384: 184–187. PMID: 8906796
12. Choe H, Farzan M, Sun Y, Sullivan N, Rollins B, et al. (1996) The beta-chemokine receptors CCR3 and CCR5 facilitate infection by primary HIV-1 isolates. Cell 85: 1135–1148. PMID: 8674119
13. Deng H, Liu R, Ellmeier W, Choe S, Unutmaz D, et al. (1996) Identification of a major co-receptor for primary isolates of HIV-1. Nature 381: 661–666. PMID: 8649511
14. Dragic T, Litwin V, Allaway GP, Martin SR, Huang Y, et al. (1996) HIV-1 entry into CD4+ cells is mediated by the chemokine receptor CC-CKR-5. Nature 381: 667–673. PMID: 8649512
15. Doranz BJ, Rucker J, Yi Y, Smyth RJ, Samson M, et al. (1996) A dual-tropic primary HIV-1 isolate that uses fusin and the beta-chemokine receptors CKR-5, CKR-3, and CKR-2b as fusion cofactors. Cell 85: 1149–1158. PMID: 8674120
16. Feng Y, Broder CC, Kennedy PE, Berger EA (1996) HIV-1 entry cofactor: functional cDNA cloning of a seven-transmembrane, G protein-coupled receptor. Science 272: 872–877. PMID: 8629022
17. Alkhatib G, Combadiere C, Broder CC, Feng Y, Kennedy PE, et al. (1996) CC CKR5: a RANTES, MIP-1alpha, MIP-1beta receptor as a fusion cofactor for macrophage-tropic HIV-1. Science 272: 1955–1958. PMID: 8658171
18. Furuta RA, Wild CT, Weng Y, Weiss CD (1998) Capture of an early fusion-active conformation of HIV-1 gp41. Nat Struct Biol 5: 276–279. PMID: 9546217
19. He Y, Vassell R, Zaitseva M, Nguyen N, Yang Z, et al. (2003) Peptides trap the human immunodeficiency virus type 1 envelope glycoprotein fusion intermediate at two sites. J Virol 77: 1666–1671. PMID: 12525600
20. Koshiba T, Chan DC (2003) The prefusogenic intermediate of HIV-1 gp41 contains exposed C-peptide regions. J Biol Chem 278: 7573–7579. PMID: 12486032
21. Si Z, Madani N, Cox JM, Chruma JJ, Klein JC, et al. (2004) Small-molecule inhibitors of HIV-1 entry block receptor-induced conformational changes in the viral envelope glycoproteins. Proc Natl Acad Sci U S A 101: 5036–5041. PMID: 15051887
22. Weng Y, Weiss CD (1998) Mutational analysis of residues in the coiled-coil domain of human immunodeficiency virus type 1 transmembrane protein gp41. J Virol 72: 9676–9682. PMID: 9811701
23. Lu M, Stoller MO, Wang S, Liu J, Fagan MB, et al. (2001) Structural and functional analysis of interhelical interactions in the human immunodeficiency virus type 1 gp41 envelope glycoprotein by alanine-scanning mutagenesis. J Virol 75: 11146–11156. PMID: 11602754
24. Mo H, Konstantinidis AK, Stewart KD, Dekhtyar T, Ng T, et al. (2004) Conserved residues in the coiled-coil pocket of human immunodeficiency virus type 1 gp41 are essential for viral replication and interhelical interaction. Virology 329: 319–327. PMID: 15518811

25. Earl PL, Broder CC, Long D, Lee SA, Peterson J, et al. (1994) Native oligomeric human immunodeficiency virus type 1 envelope glycoprotein elicits diverse monoclonal antibody reactivities. J Virol 68: 3015–3026. PMID: 7512157

26. Yang X, Farzan M, Wyatt R, Sodroski J (2000) Characterization of stable, soluble trimers containing complete ectodomains of human immunodeficiency virus type 1 envelope glycoproteins. J Virol 74: 5716–5725. PMID: 10823881

27. Yang X, Florin L, Farzan M, Kolchinsky P, Kwong PD, et al. (2000) Modifications that stabilize human immunodeficiency virus envelope glycoprotein trimers in solution. J Virol 74: 4746–4754. PMID: 10775613

28. Kovacs JM, Nkolola JP, Peng H, Cheung A, Perry J, et al. (2012) HIV-1 envelope trimer elicits more potent neutralizing antibody responses than monomeric gp120. Proc Natl Acad Sci U S A 109: 12111–12116. doi: 10.1073/pnas.1204533109 PMID: 22773820

29. Zhang CW, Chishti Y, Hussey RE, Reinherz EL (2001) Expression, purification, and characterization of recombinant HIV gp140. The gp41 ectodomain of HIV or simian immunodeficiency virus is sufficient to maintain the retroviral envelope glycoprotein as a trimer. J Biol Chem 276: 39577–39585. PMID: 11514580

30. Sanders RW, Vesanen M, Schuelke N, Master A, Schiffner L, et al. (2002) Stabilization of the soluble, cleaved, trimeric form of the envelope glycoprotein complex of human immunodeficiency virus type 1. J Virol 76: 8875–8889. PMID: 12163607

31. Sanders RW, Schiffner L, Master A, Kajumo F, Guo Y, et al. (2000) Variable-loop-deleted variants of the human immunodeficiency virus type 1 envelope glycoprotein can be stabilized by an intermolecular disulfide bond between the gp120 and gp41 subunits. J Virol 74: 5091–5100. PMID: 10799583

32. Binley JM, Sanders RW, Clas B, Schuelke N, Master A, et al. (2000) A recombinant human immunodeficiency virus type 1 envelope glycoprotein complex stabilized by an intermolecular disulfide bond between the gp120 and gp41 subunits is an antigenic mimic of the trimeric virion-associated structure. J Virol 74: 627–643. PMID: 10623724

33. Schulke N, Vesanen MS, Sanders RW, Zhu P, Lu M, et al. (2002) Oligomeric and conformational properties of a proteolytically mature, disulfide-stabilized human immunodeficiency virus type 1 gp140 envelope glycoprotein. J Virol 76: 7760–7776. PMID: 12097589

34. Sanders RW, Derking R, Cupo A, Julien JP, Yasmeen A, et al. (2013) A next-generation cleaved, soluble HIV-1 Env Trimer, BG505 SOSIP.664 gp140, expresses multiple epitopes for broadly neutralizing but not non-neutralizing antibodies. PLoS Pathog 9: e1003618. doi: 10.1371/journal.ppat.1003618 PMID: 24068931

35. Klasse PJ, Depetris RS, Pejchal R, Julien JP, Khayat R, et al. (2013) Influences on trimerization and aggregation of soluble, cleaved HIV-1 SOSIP envelope glycoprotein. J Virol 87: 9873–9885. doi: 10.1128/JVI.01226-13 PMID: 23824824

36. Lyumkis D, Julien JP, de Val N, Cupo A, Potter CS, et al. (2013) Cryo-EM Structure of a Fully Glycosylated Soluble Cleaved HIV-1 Envelope Trimer. Science.

37. Julien JP, Cupo A, Sok D, Stanfield RL, Lyumkis D, et al. (2013) Crystal structure of a soluble cleaved HIV-1 envelope trimer. Science 342: 1477–1483. doi: 10.1126/science.1245625 PMID: 24179159

38. Pancera M, Zhou T, Druz A, Georgiev IS, Soto C, et al. (2014) Structure and immune recognition of trimeric pre-fusion HIV-1 Env. Nature.

39. Bartesaghi A, Merk A, Borgnia MJ, Milne JL, Subramaniam S (2013) Prefusion structure of trimeric HIV-1 envelope glycoprotein determined by cryo-electron microscopy. Nat Struct Mol Biol 20: 1352–1357. doi: 10.1038/nsmb.2711 PMID: 24154805

40. Liu J, Bartesaghi A, Borgnia MJ, Sapiro G, Subramaniam S (2008) Molecular architecture of native HIV-1 gp120 trimers. Nature 455: 109–113. doi: 10.1038/nature07159 PMID: 18668044

41. Wu SR, Loving R, Lindqvist B, Hebert H, Koeck PJ, et al. (2010) Single-particle cryoelectron microscopy analysis reveals the HIV-1 spike as a tripod structure. Proc Natl Acad Sci U S A 107: 18844–18849. doi: 10.1073/pnas.1007227107 PMID: 20956336

42. Mao Y, Wang L, Gu C, Herschhorn A, Desormeaux A, et al. (2013) Molecular architecture of the uncleaved HIV-1 envelope glycoprotein trimer. Proc Natl Acad Sci U S A.

43. Mao Y, Wang L, Gu C, Herschhorn A, Xiang SH, et al. (2012) Subunit organization of the membrane-bound HIV-1 envelope glycoprotein trimer. Nat Struct Mol Biol.

44. Kwong PD, Wyatt R, Robinson J, Sweet RW, Sodroski J, et al. (1998) Structure of an HIV gp120 envelope glycoprotein in complex with the CD4 receptor and a neutralizing human antibody. Nature 393: 648–659. PMID: 9641677

45. LaBonte JA, Patel T, Hofmann W, Sodroski J (2000) Importance of membrane fusion mediated by human immunodeficiency virus envelope glycoproteins for lysis of primary CD4-positive T cells. J Virol 74: 10690–10698. PMID: 11044113

46. Wei X, Decker JM, Liu H, Zhang Z, Arani RB, et al. (2002) Emergence of resistant human immunodeficiency virus type 1 in patients receiving fusion inhibitor (T-20) monotherapy. Antimicrob Agents Chemother 46: 1896–1905. PMID: 12019106

47. Wu X, Parast AB, Richardson BA, Nduati R, John-Stewart G, et al. (2006) Neutralization escape variants of human immunodeficiency virus type 1 are transmitted from mother to infant. J Virol 80: 835–844. PMID: 16378985

48. Korber B, Foley BT, Kuiken C, Pillai SK, Sodroski JG (1998) Numbering Positions in HIV Relative to HXB2CG. Human Retroviruses and AIDS pp. III: 102–111.

49. Haim H, Si Z, Madani N, Wang L, Courter JR, et al. (2009) Soluble CD4 and CD4-mimetic compounds inhibit HIV-1 infection by induction of a short-lived activated state. PLoS Pathog 5: e1000360. doi: 10.1371/journal.ppat.1000360 PMID: 19343205

50. Veillette M, Coutu M, Richard J, Batraville LA, Desormeaux A, et al. (2014) Conformational Evaluation of HIV-1 Trimeric Envelope Glycoproteins Using a Cell-based ELISA Assay. J Vis Exp.

51. Rho HM, Poiesz B, Ruscetti FW, Gallo RC (1981) Characterization of the reverse transcriptase from a new retrovirus (HTLV) produced by a human cutaneous T-cell lymphoma cell line. Virology 112: 355–360. PMID: 6166122

52. Binley JM, Cayanan CS, Wiley C, Schulke N, Olson WC, et al. (2003) Redox-triggered infection by disulfide-shackled human immunodeficiency virus type 1 pseudovirions. J Virol 77: 5678–5684. PMID: 12719560

53. Abrahamyan LG, Markosyan RM, Moore JP, Cohen FS, Melikyan GB (2003) Human immunodeficiency virus type 1 Env with an intersubunit disulfide bond engages coreceptors but requires bond reduction after engagement to induce fusion. J Virol 77: 5829–5836. PMID: 12719576

54. Xiang SH, Kwong PD, Gupta R, Rizzuto CD, Casper DJ, et al. (2002) Mutagenic stabilization and/or disruption of a CD4-bound state reveals distinct conformations of the human immunodeficiency virus type 1 gp120 envelope glycoprotein. J Virol 76: 9888–9899. PMID: 12208966

55. Dey B, Pancera M, Svehla K, Shu Y, Xiang SH, et al. (2007) Characterization of human immunodeficiency virus type 1 monomeric and trimeric gp120 glycoproteins stabilized in the CD4-bound state: antigenicity, biophysics, and immunogenicity. J Virol 81: 5579–5593. PMID: 17360741

56. Kassa A, Finzi A, Pancera M, Courter JR, Smith AB 3rd, et al. (2009) Identification of a Human Immunodeficiency Virus (HIV-1) Envelope Glycoprotein Variant Resistant to Cold Inactivation. J Virol.

57. Finzi A, Xiang SH, Pacheco B, Wang L, Haight J, et al. (2010) Topological layers in the HIV-1 gp120 inner domain regulate gp41 interaction and CD4-triggered conformational transitions. Mol Cell 37: 656–667. doi: 10.1016/j.molcel.2010.02.012 PMID: 20227370

58. Desormeaux A, Coutu M, Medjahed H, Pacheco B, Herschhorn A, et al. (2013) The highly conserved layer-3 component of the HIV-1 gp120 inner domain is critical for CD4-required conformational transitions. J Virol 87: 2549–2562. doi: 10.1128/JVI.03104-12 PMID: 23255784

59. Veillette M, Desormeaux A, Medjahed H, Gharsallah NE, Coutu M, et al. (2014) Interaction with cellular CD4 exposes HIV-1 envelope epitopes targeted by antibody-dependent cell-mediated cytotoxicity. J Virol 88: 2633–2644. doi: 10.1128/JVI.03230-13 PMID: 24352444

60. Trkola A, Purtscher M, Muster T, Ballaun C, Buchacher A, et al. (1996) Human monoclonal antibody 2G12 defines a distinctive neutralization epitope on the gp120 glycoprotein of human immunodeficiency virus type 1. J Virol 70: 1100–1108. PMID: 8551569

61. Kong L, Lee JH, Doores KJ, Murin CD, Julien JP, et al. (2013) Supersite of immune vulnerability on the glycosylated face of HIV-1 envelope glycoprotein gp120. Nat Struct Mol Biol 20: 796–803. doi: 10.1038/nsmb.2594 PMID: 23708606

62. Doores KJ, Fulton Z, Huber M, Wilson IA, Burton DR (2010) Antibody 2G12 recognizes di-mannose equivalently in domain- and nondomain-exchanged forms but only binds the HIV-1 glycan shield if domain exchanged. J Virol 84: 10690–10699. doi: 10.1128/JVI.01110-10 PMID: 20702629

63. Calarese DA, Scanlan CN, Zwick MB, Deechongkit S, Mimura Y, et al. (2003) Antibody domain exchange is an immunological solution to carbohydrate cluster recognition. Science 300: 2065–2071. PMID: 12829775

64. Acharya P, Tolbert WD, Gohain N, Wu X, Yu L, et al. (2014) Structural Definition of an Antibody-Dependent Cellular Cytotoxicity Response Implicated in Reduced Risk for HIV-1 Infection. J Virol 88: 12895–12906. doi: 10.1128/JVI.02194-14 PMID: 25165110

65. Thali M, Moore JP, Furman C, Charles M, Ho DD, et al. (1993) Characterization of conserved human immunodeficiency virus type 1 gp120 neutralization epitopes exposed upon gp120-CD4 binding. J Virol 67: 3978–3988. PMID: 7685405

66. Kesavardhana S, Varadarajan R (2014) Stabilizing the native trimer of HIV-1 Env by destabilizing the heterodimeric interface of the gp41 postfusion six-helix bundle. J Virol 88: 9590–9604. doi: 10.1128/JVI.00494-14 PMID: 24920800

67. Walker LM, Phogat SK, Chan-Hui PY, Wagner D, Phung P, et al. (2009) Broad and potent neutralizing antibodies from an African donor reveal a new HIV-1 vaccine target. Science 326: 285–289. doi: 10.1126/science.1178746 PMID: 19729618

68. Walker LM, Huber M, Doores KJ, Falkowska E, Pejchal R, et al. (2011) Broad neutralization coverage of HIV by multiple highly potent antibodies. Nature 477: 466–470. doi: 10.1038/nature10373 PMID: 21849977

69. Julien JP, Sok D, Khayat R, Lee JH, Doores KJ, et al. (2013) Broadly neutralizing antibody PGT121 allosterically modulates CD4 binding via recognition of the HIV-1 gp120 V3 base and multiple surrounding glycans. PLoS Pathog 9: e1003342. doi: 10.1371/journal.ppat.1003342 PMID: 23658524

70. Huang J, Kang BH, Pancera M, Lee JH, Tong T, et al. (2014) Broad and potent HIV-1 neutralization by a human antibody that binds the gp41-gp120 interface. Nature.

71. Blattner C, Lee JH, Sliepen K, Derking R, Falkowska E, et al. (2014) Structural delineation of a quaternary, cleavage-dependent epitope at the gp41-gp120 interface on intact HIV-1 Env trimers. Immunity 40: 669–680. doi: 10.1016/j.immuni.2014.04.008 PMID: 24768348

72. Falkowska E, Le KM, Ramos A, Doores KJ, Lee JH, et al. (2014) Broadly neutralizing HIV antibodies define a glycan-dependent epitope on the prefusion conformation of gp41 on cleaved envelope trimers. Immunity 40: 657–668. doi: 10.1016/j.immuni.2014.04.009 PMID: 24768347

73. Wyss S, Dimitrov AS, Baribaud F, Edwards TG, Blumenthal R, et al. (2005) Regulation of human immunodeficiency virus type 1 envelope glycoprotein fusion by a membrane-interactive domain in the gp41 cytoplasmic tail. J Virol 79: 12231–12241. PMID: 16160149

74. Edwards TG, Wyss S, Reeves JD, Zolla-Pazner S, Hoxie JA, et al. (2002) Truncation of the cytoplasmic domain induces exposure of conserved regions in the ectodomain of human immunodeficiency virus type 1 envelope protein. J Virol 76: 2683–2691. PMID: 11861835

75. Abrahamyan LG, Mkrtchyan SR, Binley J, Lu M, Melikyan GB, et al. (2005) The cytoplasmic tail slows the folding of human immunodeficiency virus type 1 Env from a late prebundle configuration into the six-helix bundle. J Virol 79: 106–115. PMID: 15596806

76. Haim H, Salas I, McGee K, Eichelberger N, Winter E, et al. (2013) Modeling virus- and antibody-specific factors to predict human immunodeficiency virus neutralization efficiency. Cell Host Microbe 14: 547–558. doi: 10.1016/j.chom.2013.10.006 PMID: 24237700

77. Haim H, Strack B, Kassa A, Madani N, Wang L, et al. (2011) Contribution of Intrinsic Reactivity of the HIV-1 Envelope Glycoproteins to CD4-Independent Infection and Global Inhibitor Sensitivity. PLoS Pathog 7: e1002101. doi: 10.1371/journal.ppat.1002101 PMID: 21731494

78. Julien JP, Lee JH, Cupo A, Murin CD, Derking R, et al. (2013) Asymmetric recognition of the HIV-1 trimer by broadly neutralizing antibody PG9. Proc Natl Acad Sci U S A 110: 4351–4356. doi: 10.1073/pnas.1217537110 PMID: 23426631

79. Doria-Rose NA, Schramm CA, Gorman J, Moore PL, Bhiman JN, et al. (2014) Developmental pathway for potent V1V2-directed HIV-neutralizing antibodies. Nature.

80. Murin CD, Julien JP, Sok D, Stanfield RL, Khayat R, et al. (2014) Structure of 2G12 Fab2 in complex with soluble and fully glycosylated HIV-1 Env by negative-stain single-particle electron microscopy. J Virol 88: 10177–10188. doi: 10.1128/JVI.01229-14 PMID: 24965454

81. Chen L, Kwon YD, Zhou T, Wu X, O'Dell S, et al. (2009) Structural basis of immune evasion at the site of CD4 attachment on HIV-1 gp120. Science 326: 1123–1127. doi: 10.1126/science.1175868 PMID: 19965434

82. Chakrabarti BK, Pancera M, Phogat S, O'Dell S, McKee K, et al. (2011) HIV type 1 Env precursor cleavage state affects recognition by both neutralizing and nonneutralizing gp41 antibodies. AIDS Res Hum Retroviruses 27: 877–887. doi: 10.1089/AID.2010.0281 PMID: 21158699

83. Zhou T, Georgiev I, Wu X, Yang ZY, Dai K, et al. (2010) Structural basis for broad and potent neutralization of HIV-1 by antibody VRC01. Science 329: 811–817. doi: 10.1126/science.1192819 PMID: 20616231

84. Sok D, Doores KJ, Briney B, Le KM, Saye-Francisco KL, et al. (2014) Promiscuous glycan site recognition by antibodies to the high-mannose patch of gp120 broadens neutralization of HIV. Sci Transl Med 6: 236ra263.

85. Mouquet H, Scharf L, Euler Z, Liu Y, Eden C, et al. (2012) Complex-type N-glycan recognition by potent broadly neutralizing HIV antibodies. Proc Natl Acad Sci U S A 109: E3268–3277. doi: 10.1073/pnas.1217207109 PMID: 23115339

86. Sen J, Yan T, Wang J, Rong L, Tao L, et al. (2010) Alanine scanning mutagenesis of HIV-1 gp41 heptad repeat 1: insight into the gp120-gp41 interaction. Biochemistry 49: 5057–5065. doi: 10.1021/bi1005267 PMID: 20481578

87. Sanders RW, Busser E, Moore JP, Lu M, Berkhout B (2004) Evolutionary repair of HIV type 1 gp41 with a kink in the N-terminal helix leads to restoration of the six-helix bundle structure. AIDS Res Hum Retroviruses 20: 742–749. PMID: 15307920

88. Kwon YD, Finzi A, Wu X, Dogo-Isonagie C, Lee LK, et al. (2012) Unliganded HIV-1 gp120 core structures assume the CD4-bound conformation with regulation by quaternary interactions and variable loops. Proc Natl Acad Sci U S A 109: 5663–5668. doi: 10.1073/pnas.1112391109 PMID: 22451932

89. Kassa A, Madani N, Schon A, Haim H, Finzi A, et al. (2009) Transitions to and from the CD4-bound conformation are modulated by a single-residue change in the human immunodeficiency virus type 1 gp120 inner domain. J Virol 83: 8364–8378. doi: 10.1128/JVI.00594-09 PMID: 19535453

90. Yang X, Mahony E, Holm GH, Kassa A, Sodroski J (2003) Role of the gp120 inner domain beta-sandwich in the interaction between the human immunodeficiency virus envelope glycoprotein subunits. Virology 313: 117–125. PMID: 12951026

91. Goddard TD, Huang CC, Ferrin TE (2007) Visualizing density maps with UCSF Chimera. J Struct Biol 157: 281–287. PMID: 16963278

92. Rossmann MG, Bernal R, Pletnev SV (2001) Combining electron microscopic with x-ray crystallographic structures. J Struct Biol 136: 190–200. PMID: 12051899

93. Pettersen EF, Goddard TD, Huang CC, Couch GS, Greenblatt DM, et al. (2004) UCSF Chimera—a visualization system for exploratory research and analysis. J Comput Chem 25: 1605–1612. PMID: 15264254
